# Hydrodynamic stress maps on the surface of a flexible fin-like foil

**DOI:** 10.1371/journal.pone.0244674

**Published:** 2021-01-12

**Authors:** Paule Dagenais, Christof M. Aegerter

**Affiliations:** Physik-Institut, University of Zurich, Zurich, Switzerland; Coastal Carolina University, UNITED STATES

## Abstract

We determine the time dependence of pressure and shear stress distributions on the surface of a pitching and deforming hydrofoil from measurements of the three dimensional flow field. Period-averaged stress maps are obtained both in the presence and absence of steady flow around the foil. The velocity vector field is determined via volumetric three-component particle tracking velocimetry and subsequently inserted into the Navier-Stokes equation to calculate the total hydrodynamic stress tensor. In addition, we also present a careful error analysis of such measurements, showing that local evaluations of stress distributions are possible. The consistency of the force time-dependence is verified using a control volume analysis. The flapping foil used in the experiments is designed to allow comparison with a small trapezoidal fish fin, in terms of the scaling laws that govern the oscillatory flow regime. As a complementary approach, unsteady Euler-Bernoulli beam theory is employed to derive instantaneous transversal force distributions on the flexible hydrofoil from its deflection and the results are compared to the spatial distributions of hydrodynamic stresses obtained from the fluid velocity field.

## 1 Introduction

The evaluation of hydrodynamic forces on the surface of submerged and deforming foils constitutes a key element in understanding how synthetic and animal fins interact with the surrounding fluid to achieve their appropriate functions of propulsion and maneuvering. Great effort is deployed by scientists to describe the motion of fish fins and explore the physics underlying their complex kinematics [1–17]. An experimental workflow is proposed here to measure local distributions of hydrodynamic pressure and viscous stress over the surfaces of a flexible artificial fin, starting from the volumetric acquisition of 3D flow velocimetry data. This methodology broadens the possibility of studying in detail the propulsion and force regulating mechanisms in fish or biomimetic systems [18].

The desire to reproduce the elegant complexity of nature has inspired the fabrication of elaborate fins models [19–24]. Countless studies have resorted to particle imaging or particle tracking velocimetry (PIV and PTV) to investigate the flow topology and hydrodynamic forces of bio-inspired or authentic fish fins [25–37]. Euler-Bernoulli beam theory has also been employed to describe the fluid-structure interactions of fins and hydrofoils, addressing concepts such as foil compliance, damping effects, resonance frequency and efficiency optimization [38–43].

Due to the fundamental three-dimensional complexity of fin kinematics and wake structures, the empirical investigation of their hydrodynamics must escape the limitations of planar PIV/PTV [44, 45]. Given the three-dimensionality of the wake, it still remains a big challenge to determine the propulsive forces on the moving appendages. One technique consists in determining the momentum deficit in the wake, based on the vorticity field [4, 7, 11, 46–49], where the concept of vortex added-mass should also be included in a complete depiction of wake momentum exchanges [50–53]. An alternative approach to calculate the total hydrodynamic force relies on a momentum balance inside a control volume and was also employed in the context of flapping fin-like structures [26, 27, 35, 54–66]. A non-invasive method to determine the three-dimensional pressure field inside an unsteady flow domain consists in solving the Navier-Stokes equation, in either one of the two following forms: (1) the pressure gradient is expressed in terms of spatial and temporal derivatives of the velocity field and engaged in a direct spatial integration to compute the pressure values everywhere in the domain [17, 66–84], or (2) the Laplacian of the pressure is obtained by taking the divergence of the latter pressure gradient formulation, and this so-called Poisson equation is solved numerically [70, 73, 74, 81, 85–102]. In both cases, prescribing appropriate boundary conditions constitutes a crucial step in the calculation. An exhaustive review of theoretical and historical aspects of pressure calculations based on the velocity field is presented in [103]. Recently, a detailed analysis of error propagation in the omni-directional method for the integration of the pressure gradient field was presented in [104]. Omni-directional approaches offer high levels of accuracy in the resulting pressure field [69, 75, 84, 105, 106]. Alternatively, reducing experimental errors in raw fluid velocity fields prior to integrating the pressure gradient can result in a significant improvement of the pressure field accuracy even when using a low number of integration paths such as in the case of the eight-paths integration scheme of [77], as demonstrated in [107] with an irrotation correction method.

Besides, several groups have resorted to PIV/PTV methods to resolve the laminar and turbulent shear layers forming over solid surfaces moving in water, such as undulating membranes [94] and fish [108]. Wall shear stress determination based on PIV was also conducted in small arteries models (diameter below 1 cm) with blood mimicking fluid [109]. Two important difficulties arise in the evaluation of wall shear stress from the fluid velocity field: (1) the ratio of boundary layer thickness to spatial resolution must be large enough to allow the estimation of the velocity gradients inside that layer and (2) the stress profiles must be measured as close as possible to the real surface, where reflections can hinder the detection of particles. The shear stress profile remains fairly well captured (with a slight offset in magnitude) as long as the reconstructed surface stands sufficiently close to the real object (distance below 1 mm according to [110]). Using volumetric PTV experiments, we recently showed the feasibility of resolving the velocity field over a rigid plate pitching inside a water tunnel, with a spatial resolution of 0.75 mm, and we measured the thickness of the boundary layer within a dynamic range of about [2, 6] mm [111].

In the present work, we apply a similar methodology to resolve the shear forces over the surface of a flexible hydrofoil flapping in comparable flow regimes, combined with the pressure gradient integration method. Thus, we demonstrate the possibility to obtain well resolved distributions of pressure and viscous shear stresses, both spatially and temporally, on the surface of a synthetic fin whose size is of the order of 1 cm^2^. For this purpose, we apply a full volumetric, three components particle tracking velocimetry technique (3D-3C PTV) for the first time in this context. In our study, special attention is paid to the time and spatial resolutions and their experimental limitations, and we present a careful analysis of the error propagation from the tracked particle positions to the final hydrodynamic stress values. This work demonstrates the feasibility of experimentally capturing the full 3D force field in the fluid environment of a flapping flexible foil with dimensions comparable to that of a small fish, and to employ a force decomposition method to project the hydrodynamic stress tensor on the relevant morphological axes of the fin.

As a complementary calculation, we use the unsteady Euler-Bernoulli beam deflection theory to derive the instantaneous transversal force distributions acting on the deflecting hydrofoil. This approach relies on the assumptions of small angle deflections and rigid, planar cross-sections, remaining perpendicular to the deformed midline, considering deflections in one plane only [112]. Therefore, it offers a more approximative framework to describe fluid forces acting along the foil, as compared to the hydrodynamic forces derived from the 3D flow field. Nevertheless, both methods have been employed in previous work to study flexible foils flapping inside a fluid, and it is interesting to compare their results for the same experiment.

The rest of the paper is organized as follows. In section 2.1, the basic principles behind the experimental setup are described, before the characteristics of the model fin and the hydrodynamic stress calculation are introduced in the subsequent sections. In order to calculate forces on the respective fin surfaces from the stress tensor, the surface needs to be parameterized, which is described in section 2.4. The description is continued with the basis of alternative methods, such as Euler-Bernoulli theory and a control volume analysis, before the materials and methods section is closed with a description of the limitations in uncertainty and resolutions. Section 3.1 shows the main results in the determination of the hydrodynamic stress tensor for two different experimental situations, which are validated in a control volume analysis in section 3.2 and compared to results from Euler-Bernoulli theory in section 3.3. Finally, the limits of the method are presented in terms of the uncertainties for the obtained forces and how they relate to different flow structures in section 3.4. The results are put into context with possible applications in the discussion and outlook.

## 2 Material and methods

### 2.1 Volumetric particle tracking velocimetry

PIV and PTV techniques have become widespread in the past decades in various hydrodynamics research fields and have been extensively described in the literature [113–116]. We performed volumetric three-components PTV measurements using the V3V-9800 system (TSI Incorporated, 500 Cardigan Road, Shoreview, Minnesota 55126 USA), which is well characterized in [117]. This method presents the advantage of capturing the full unsteady flow field in a single recording of the whole 3D domain, and thus is preferable to more arduous implementations of scanning stereo-PIV [63, 95, 118], where a large number of measurement planes is required to reconstruct the whole volumetric flow field, or tomographic PIV, where the whole volume needs to be scanned with a light sheet for each particle position field, which is challenging with a flexible membrane deforming rapidly in the flow [66, 119].

**Fig 1:**
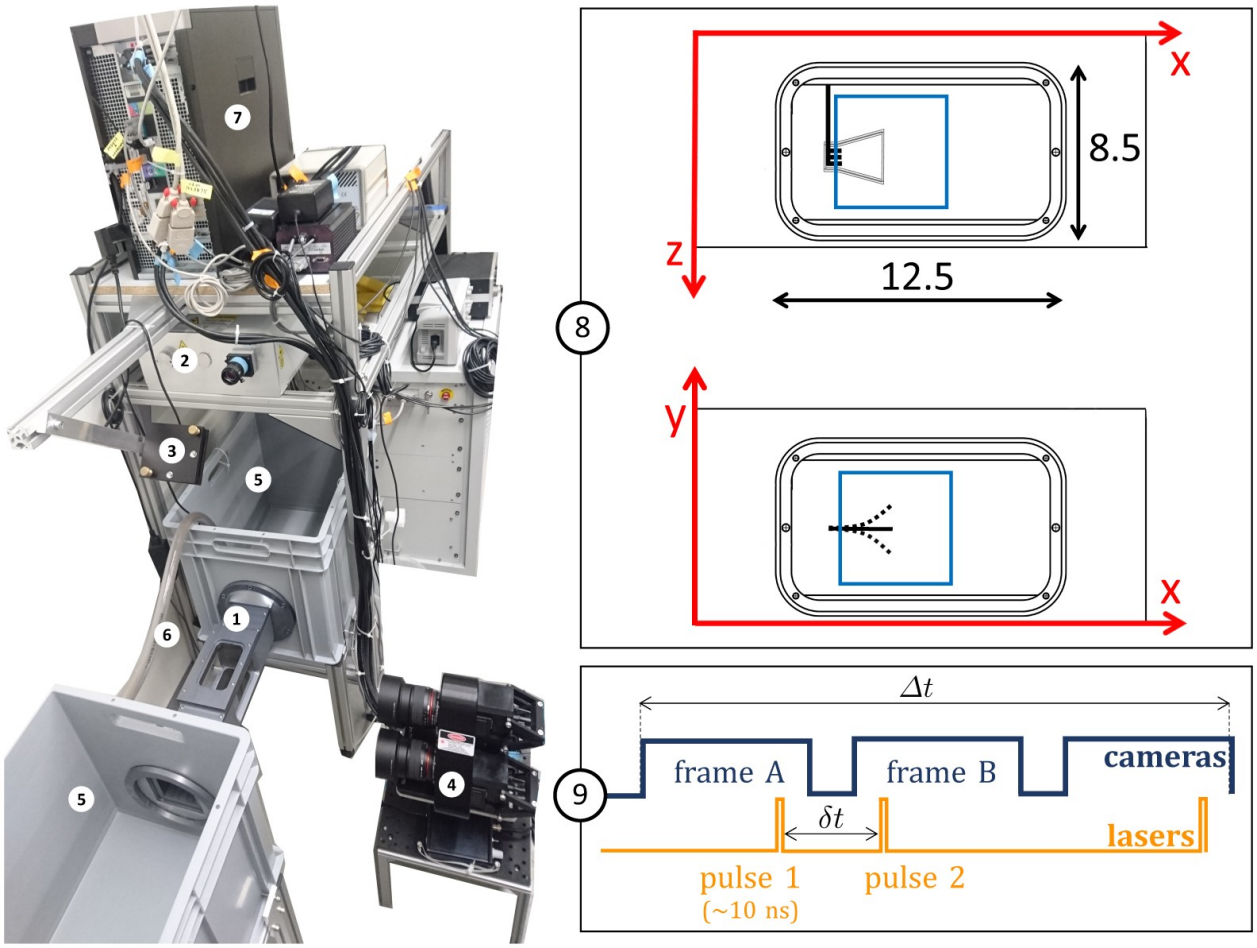
Experimental setup for 3D-3C PTV. (1) Flow chamber with transparent windows on three sides and a fixation wall on one side for inserting the synthetic fin. (2) Dual-head pulsed Nd:YAG laser with wave-length of 532 nm and maximal energy of 120 mJ/pulse, equipped with a pair of cylindrical lenses to expand the beam. (3) Mirror to deflect the laser beam. (4) Three cameras (resolution 4 MP, 85 mm lenses, sensor size 11.3×11.3 mm^2^, magnification 0.3, maximal frequency of capture 180 Hz) mounted on a triangular plate parallel to the front wall of the flow chamber (*x*-*y* plane). The distance between the cameras plate and the center of the water tunnel is ∼465 mm. (5) Water tanks for the recirculating system. (6) Pipe and pump to control the flow. (7) V3V software and synchronizer. (8) Close-up of the flow chamber with inner dimensions (in cm), PTV-interrogation volume in blue (∼50×50×20 mm^3^) and sketch of the trapezoidal fin. Frontal view (*x*−*y* plane) and top view (*x*−*z* plane). (9) Frame straddling method with *δt* between the particle images and Δ*t* between the velocity fields.

In the present setup, illustrated in Fig 1, a synthetic fin is attached to a small metallic rod and inserted through the back wall of the flow chamber, with a servomotor fixed outside the water tunnel to activate the pitching program of the rotation axis. Double pulses emitted by a dual laser system illuminate a measurement volume (50×50×20 mm^3^) inside the flow chamber. The working fluid is water and the seeding tracers are polyimide particles with a diameter of ∼50 *μ*m, which were used in a precedent study of fish locomotion based on particle velocimetry [15]. In total, ∼45 mL of particles are seeded in ∼160 L of water, which yields an approximate number of 8×10^4^ particles inside the interrogation volume, considering that the spherical particles powder has a filling fraction of ∼40%. Three cameras mounted on a triangular plate are recording triplets of images. Hence, the tracer particles advected by the flow are captured from three different perspectives at each time point. Each of the three images is analyzed based on a 2D Gaussian fit of the particle intensity distribution to identify the 2D position map. The 3D field of particle positions is reconstructed by overlapping the three viewing angles based on a triplet search algorithm. Flow velocities are determined based on individual particle displacements in the time between subsequent laser pulses (*δt* = 2.5 ms), using a relaxation method to achieve a probability-based matching. Appropriate synchronization is arranged between the cameras and lasers in a temporal scheme called frame straddling, as illustrated in Fig 1. The rate at which velocity fields are recorded (80 Hz) is half the camera acquisition frequency, yielding a time separation of Δ*t* = 0.0125 s between the velocity fields. This allows the reconstruction of the material acceleration field, necessary for the hydrodynamic stress calculation.

The image post-processing was conducted using the V3V software (version 2.0.2.7) along with an optimal set of parameters, selected to reduce the number of velocity outliers while preserving the main flow features. The maximum overlap between two particle intensity distributions was set to 65%, a mask was applied over the foil during the particle identification step, and appropriate median filter and velocity range (±0.25 m/s) were applied to the raw velocity fields. Finally, the particle velocities were interpolated on a regular 3D grid using Gaussian interpolation, yielding a final spatial grid spacing of 0.75 mm in each direction. Precedent studies found that appropriate smoothing of the velocity fields could reduce the error in pressure fields produced by the *queen2* algorithm from [77] by 30-67% [80, 107]. Precautions need to be taken for turbulent flow fields to avoid smoothing out high frequency flow structures which could compromise the pressure field reconstruction. In this case, a moderate Gaussian smoothing scheme with a factor of 1.5 (corresponding to the ratio between the Gaussian radius and the voxel half size) was applied to the interpolated velocity field. The grid points inside the hydrofoil boundary, where no particle is detected owing to the mask, are not attributed a velocity value and are not involved in the hydrodynamic stress calculation (see section 2.4).

### 2.2 Geometry and kinematics of the synthetic fin

**Fig 2:**
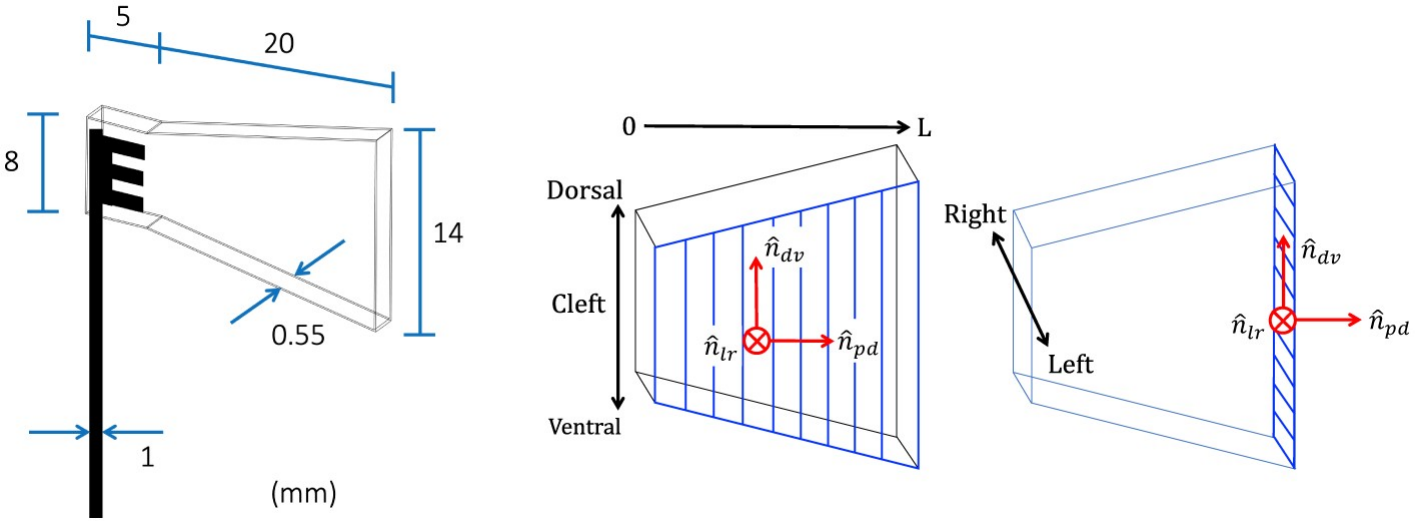
*Left*: Synthetic fin dimensions. *Right*: Main axes on the trapezoidal fin (proximo-distal, dorso-ventral, left-right).

A synthetic trapezoidal fin was designed with proportions reminiscent of the caudal fin of a small fish. The dimensions of the fin model are shown in Fig 2 and the experimental parameters are listed in Table 1. The flexible foil was produced by 3D printing a rigid cast of its negative form, then pouring liquid PDMS (polydimethylsiloxane) inside the cast, installing the fork-shaped base rod at the appropriate location, and curing the model for 36 hours at 58°C. This resulted in a flexible membrane of thickness 0.55 mm. The mass density fo the foil *ρ*_*f*_ matches that of water (http://www.mit.edu/~6.777/matprops/pdms.htm). A study of elastic properties of real caudal fins has reported an effective Young’s modulus of ∼8 MPa for the zebrafish, including both the rays and interray tissue contributions [120, 121]. The same cantilever deflection setup was employed by Sahil Puri (University of Zurich) to characterize the elastic properties of our PDMS foil, resulting in a value of ∼0.8 MPa. In order to further characterize the properties of our fin model, we performed a mechanical damping analysis, where the fin was simply held from the tip at a certain amplitude and released (in air and in water). The time evolution of the tip amplitude was modeled by a decaying oscillation:

$$\begin{array}{c} y\left(t\right)=e^{-\gamma t}Acos\left(2\pi f_{0}t-\phi \right) \end{array}$$ (1)

**Table 1:** Parameters of the oscillating fin model for the two experimental cases.

	Exp. 1	Exp. 2
*u*_∞_ (mm/s)	0	17
*θ*_0_ (°)	10	25
*a* (mm)	11	14
*f* (Hz)	3	3
*L* (mm)	25	25
${\vec{u}}_{ave}$ (mm/s)	15.4	29.0
*Re*	385	725
*St*	2.1	1.5

We found values of *γ*_*air*_ = 1.5 s^-1^ and *γ*_*water*_ = 5.0 s^-1^ for the decay rates in air and in water, respectively, and values of *f*_0,*air*_ = 9.3 Hz and *f*_0,*water*_ = 2.4 Hz for the eigenfrequencies. These results are needed for the estimation of the damping terms in the Euler-Bernoulli equation (see section 2.5).

Two versions of the pitching fin experiment were conducted, with different pitching amplitude *θ*_0_ and upstream velocity *u*_∞_ (Table 1). The dimensionless Reynolds and Strouhal numbers are employed to characterize the flow regime of the flapping foil:

$$\begin{array}{c} Re=\frac{U\cdot L}{\nu }\;St=\frac{f\cdot a}{U} \end{array}$$ (2)

These parameters are based on the kinematic viscosity of water (*ν*), the fluid velocity averaged over the whole measurement volume and a complete flapping cycle (${\vec{u}}_{ave}$), the foil length (*L* = 25 mm), the flapping frequency (*f* = 3 Hz) and the tip-to-tip excursion amplitude (*a*, obtained from the parametrized deflection profiles—see section 2.4). Even within a single fish species, a wide range of *St* and *Re* can be reached by caudal fin motion, depending on the developmental stage (larval, juvenile or adult) and the swimming behavior. For example, zebrafish undergoes a developmental transition which allows it to cover a large spectrum of Reynolds and Strouhal numbers throughout its lifetime, ranging from 390 to 2600 in *Re* (using the caudal fin length to allow direct comparison with isolated hydrofoils) and from 0.3 to 2.5 in *St*, based on swimming speed, tail beat frequency and amplitude reported by numerous studies [14, 17, 122–129]. The Reynolds numbers of the two experimental cases presented here (385 and 725), although not representative of maximal velocity in fish locomotion, correspond to slow cruising regimes characteristic of certain species [130] and specific behaviors such as foraging, chemotaxis and exploration. The chosen flapping frequency (3 Hz) is also comparable to typical tail-beat frequency in several fish species [131–134]. Moreover, the frequency was selected in the vicinity of the resonance frequency of the foil-fluid system (*f*_0_ = 2.4 Hz). In addition to the well known Strouhal number efficiency window of (0.2-0.4) [135, 136], it is generally accepted that a driving frequency close to the eigenfrequency of the oscillator will result in improved efficiency in thrust production [38, 65, 137–141]. Therefore, experiments with flexible foils flapping close to the resonance frequency are of particular relevance for the field of underwater propulsion and fish locomotion. The frequency chosen for our experiments correspond to a flexural frequency of 1.25 (defined as f* = *f*/*f*_0_). The flexural rigidity f* characterizes the relative time scale of the flapping motion with respect to the time scale of the fluid-structure resonance. It can be used to describe the fluid-structure interaction, similarly to the Strouhal number which characterizes the relative time scales of flapping behavior with respect to the external flow [141]. Therefore, although not yielding values of *St* typically related to high propulsion efficiency, the chosen value of the flapping frequency is pertinent for hydrodynamic problems concerned with the behavior of flexible structures oscillating near resonance, and is comparable to values employed in other studies of fin-like hydrofoils [80, 137, 139, 141–146]. The experimental cases presented here are not designed to achieve high propulsive efficiency, rather, to demonstrate the feasibility of obtaining well resolved stress maps on all fin surfaces. This methodology could be applied to other ranges of *St* and *Re* to study problems concerned with specific flow regimes, for example, by tuning the external incoming flow velocity or the flapping frequency, as shown in a recent study [18].

### 2.3 Hydrodynamic stress calculation

The starting point of the hydrodynamic stress calculation is the Navier-Stokes or momentum equation [147, 148]:

$$\begin{array}{c} \rho \frac{D\vec{u}}{Dt}=\vec{\nabla }\cdot s+\rho \vec{g} \end{array}$$ (3)

The last term on the right corresponds to any type of body force acting on the fluid, such as gravity which can be measured independently of the flow. This can be included in a static pressure term and we will omit it in the following. The left side of the equation contains the material acceleration, with the Lagrangian derivative *D*/*Dt*. The first term on the right side is the divergence of the total hydrodynamic stress tensor *s*_*ij*_ = −*pδ*_*ij*_ + *τ*_*ij*_, where *p* is the scalar pressure field and ***τ*** the viscous stress tensor. This viscous shear stress can be expressed as:

$$\begin{array}{c} \tau_{ij}=\mu (\frac{\partial u_{i}}{\partial x_{j}}+\frac{\partial u_{j}}{\partial x_{i}}), \end{array}$$ (4)

where *μ* is the dynamic viscosity. Computationally, *τ*_*ij*_ can be obtained directly using centered finite differences between neighboring velocity vectors:

$$\begin{array}{c} \begin{array}{cc} \tau_{ij}≃ & \mu (\frac{u_{i}\left(\vec{x}+\Delta x\hat{j}\right)-u_{i}\left(\vec{x}-\Delta x\hat{j}\right)}{2\Delta x}\\ & +\frac{u_{j}\left(\vec{x}+\Delta x\hat{i}\right)-u_{j}\left(\vec{x}-\Delta x\hat{i}\right)}{2\Delta x}+O\left(\Delta x^{2}\right)) \end{array} \end{array}$$ (5)

In order to determine the pressure from the flow fields, we first rewrite the Navier-Stokes equation to isolate the pressure gradient on one side:

$$\begin{array}{c} \begin{array}{cc} \frac{\partial p}{\partial x_{i}}= & -\rho (\frac{\partial u_{i}}{\partial t}+u_{i}\frac{\partial u_{i}}{\partial x_{i}}+u_{j}\frac{\partial u_{i}}{\partial x_{j}})+\mu (\frac{\partial^{2}u_{i}}{\partial x_{i}^{2}}+\frac{\partial^{2}u_{i}}{\partial x_{j}^{2}})\\ & +(-\rho u_{k}\frac{\partial u_{i}}{\partial x_{k}}+\mu \frac{\partial^{2}u_{i}}{\partial x_{k}^{2}}) \end{array} \end{array}$$ (6)

The pressure field can then be reconstructed by integrating this equation, with the additional requirement of specifying the pressure values at the starting points of the integration paths $p({\vec{r}}_{ref})$.

The pressure calculation was conducted using the *queen2* algorithm [77], which is available at http://dabirilab.com/software/. The calculation assumes zero-pressure values on the external boundary of the domain, in the undisturbed flow, and integrates the pressure gradient along eight different paths (horizontal, vertical or diagonal) originating from the outside contour, among which the median is selected for each node in the 3D domain. The object is masked to prevent any integration path crossing its boundary. This algorithm offers the advantage of reasonable computation times even for large domains, with a total number of paths equal to 8 times the domain size (*N*_*x*_ × *N*_*y*_ × *N*_*z*_) per velocity field, resulting in a more than order-of-magnitude improvement in computation time compared to other multi-path integration schemes. Omni-directional schemes can provide higher accuracy [69, 75, 84, 105] but for 3D implementation, the use of GPU-based calculation was demonstrated to achieve accelerated computational times [106]. The terms containing out-of-plane derivatives of the velocity (grouped separately in Eq 6) are not included directly in the computation. Rather, the three-dimensionality is handled through a combination of integration paths originating from all the faces of the domain, combined with a median polling. More details on how this median polling approach allows to reduce the accumulation of measurement errors are provided in [77].

The central step of the pressure calculation consists in evaluating the material acceleration, which can be done in the Eulerian or the Lagrangian frame. The Eulerian frame is stationary in the laboratory, where the time derivative of the velocity is expressed as a total derivative:

$$\begin{array}{c} \frac{D}{Dt}=\frac{\partial }{\partial t}+\frac{\partial x_{i}}{\partial t}\cdot \frac{\partial }{\partial x_{i}}=\frac{\partial }{\partial t}+\vec{u}\cdot \vec{\nabla } \end{array}$$ (7)

**Fig 3:**
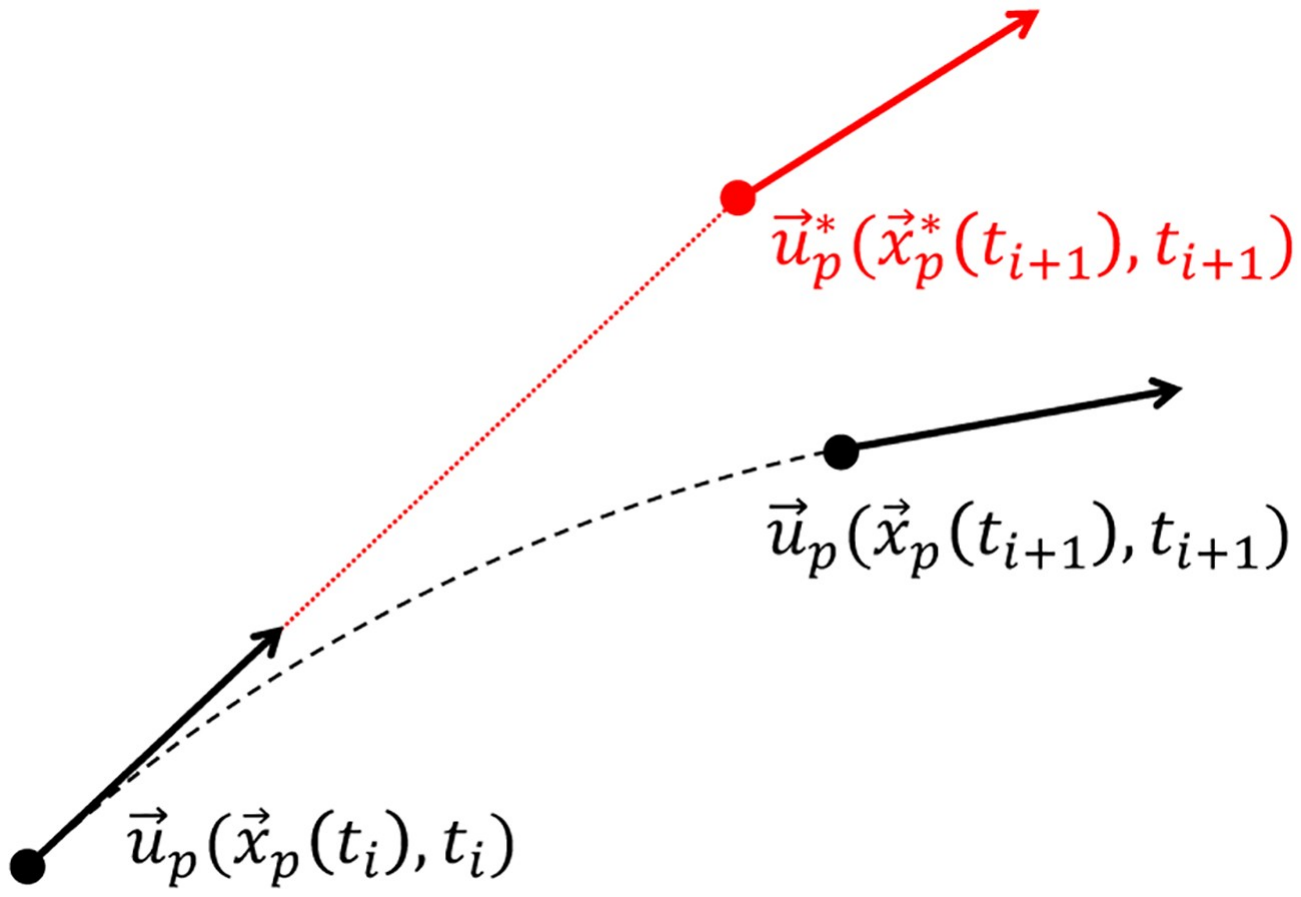
Lagrangian scheme for the material acceleration calculation: True trajectory (in black) versus reconstructed trajectory (in red).

In the *queen2* algorithm, the material acceleration is evaluated based on a Lagrangian forward scheme (Fig 3), which consists in following a particle trajectory in a pseudo-tracking formulation:

$$\begin{array}{c} \frac{D{\vec{u}}_{p}}{Dt}\left({\vec{x}}_{p}\left(t_{i}\right),t_{i}\right)≃\frac{{\vec{u}}_{p}^{*}\left({\vec{x}}_{p}^{*}\left(t_{i+1}\right),t_{i+1}\right)-{\vec{u}}_{p}\left({\vec{x}}_{p}\left(t_{i}\right),t_{i}\right)}{\Delta t} \end{array}$$ (8)

The particle velocity ${\vec{u}}_{p}^{*}\left({\vec{x}}_{p}^{*}\left(t_{i+1}\right),t_{i+1}\right)$ at a forward instant relies on a linear reconstruction of the particle path to determine its position ${\vec{x}}_{p}^{*}\left(t_{i+1}\right)$, based on a trapezoidal scheme:

$$\begin{array}{c} {\vec{x}}_{p}^{*}\left(t_{i+1}\right)≃{\vec{x}}_{p}\left(t_{i}\right)+(\frac{{\vec{u}}_{p}\left({\vec{x}}_{p}\left(t_{i}\right),t_{i}\right)+{\vec{u}}_{p}\left({\vec{x}}_{p}\left(t_{i+1}\right),t_{i+1}\right)}{2})\Delta t \end{array}$$ (9)

An improvement of the pseudo-tracking approach was proposed in [69], relying on two cameras to obtain combinations of four staggered exposures of the particle traces in order to determine the Lagrangian velocity. The simplified Lagrangian scheme from [77] is used instead, which was shown to provide accurate time-resolved pressure contours for different numerical and experimental cases, including a simulated 3D anguilliform swimmer [77], and most relevant for the present study, a flexible foil flapping inside a water tunnel, where the total forces on the foil compared favorably with load cells measurements [80].

Two types of stress maps are extracted from the flow field: instantaneous distributions and period-averaged distributions, *i.e*. averaged over equidistant time points across a full motion cycle. In experiment 1, eight pairs of velocity fields covering a complete period are selected for the calculation of instantaneous stresses. As a second step, the stress tensor is projected on the fin surfaces, and the forces are decomposed into biologically relevant components (along the fin principal axes—see Fig 2). The normal stress (in absolute value) is averaged for these eight time points to obtain the period-averaged hydrodynamic load over the fin. In experiment 2, the period-averaged normal stress (in absolute value) is presented to offer a comparison with the results from experiment 1. This allows to observe which portions of the fin surface are subjected to the highest normal loads, in average during a full cycle, and to determine how this distribution changes with or without an external current (experiment 1 versus experiment 2).

### 2.4 Surface parametrization

**Fig 4:**
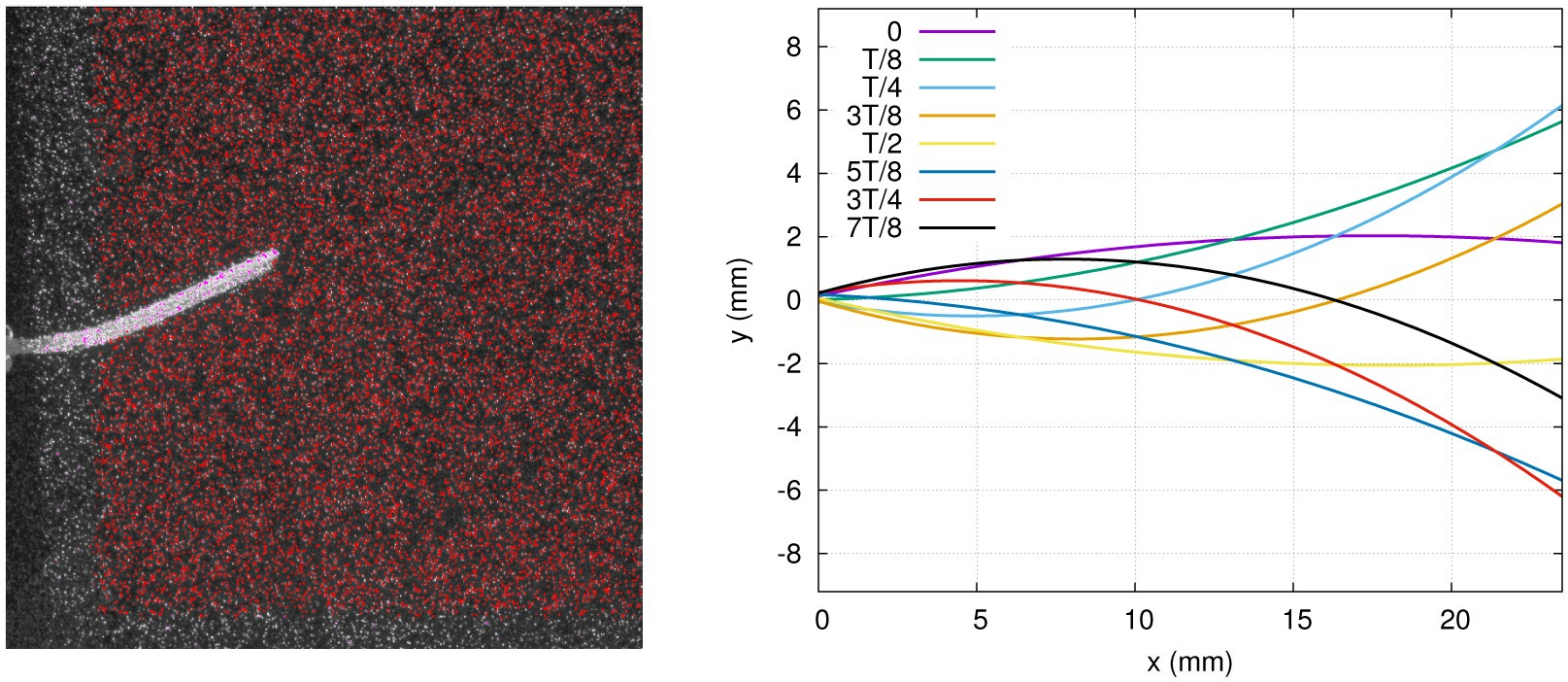
*Left*: **3D** reconstructed particle positions (red points) projected on a single raw image from the triplet. *Right*: Foil midline positions in exp. 1, fitted with a polynomial of degree 2.

The raw images offer a very clear view of the foil midline curvature (see left panel of Fig 4 for example), and are used to parametrize the midline position with a polynomial of degree 2. For each instantaneous time frame under study, 25 points are manually superimposed on the fin, allowing to fit the quadratic function to the hydrofoil time-dependent deflection. The 3D hydrofoil, including the left and right trapezoidal sides plus the tip surface, is reconstructed based on the parametrized midline, assuming that deflection occurs only along one axis. This is a reasonable assumption since the foil length is about twice as large as its width. The virtual surface reconstructed from the parameterized deflection is located at a small distance from the real foil (0.725 mm) owing to the fact that the particles can not be resolved directly at the surface due to reflections (see the masked region around the fin in Figs 4 and 7 for example).

A crucial step in our analysis is the projection of the total hydrodynamic stress tensor onto the different surfaces of the foil. The *i*-component of the stress (force per unit area) acting on a surface with a unit normal vector $\hat{n}$ (oriented outwards) is expressed as:

$$\begin{array}{c} S_{i}\left(\vec{x},t,\hat{n}\right)=s_{ij}\left(\vec{x},t\right)\cdot n_{j} \end{array}$$ (10)

The stress vector is initially expressed in cartesian coordinates. It is then decomposed into more relevant components, related to a set of orthogonal axes moving along with each fin surface, as illustrated in Fig 2. Anticipating useful analogies between our fin model and a real caudal fin, we employ the following terminology to define this coordinate system: the dorso-ventral axis ${\hat{n}}_{dv}$, the proximo-distal axis ${\hat{n}}_{pd}$ and the left-right axis ${\hat{n}}_{lr}$. These orthogonal sets are defined locally everywhere on the foil surfaces (sides and tip) and travel with it during the motion cycle. Thus, each grid point on the foil surfaces (25 nodes along the dorso-ventral axis, 25 nodes along the proximo-distal axis, 3 nodes along the left-right axis) is associated with a local set of perpendicular unit vectors (${\hat{n}}_{pd}$, ${\hat{n}}_{lr}$, ${\hat{n}}_{dv}$), on which the stress vector is projected to give *S*_*pd*_, *S*_*lr*_ and *S*_*dv*_ respectively. The pressure contribution to the total stress is always automatically acting along the normal direction onto the local surface.

In the present experimental cases, the fin operates in the inertial flow regime (although theoretically very close to the transitional range 300<*Re*<1000 [149]), where the normal stress component (dominated by the pressure) is typically larger than the viscous tangential stresses by at least two orders of magnitude. Depending on the flow under investigation, these distinct stress components may have different relative magnitudes. For example, the fish larvae evolve in a flow regime where the viscous shear stress is substantially larger [149]. Moreover, the tangential shear stress may have a particular significance in certain hydrodynamic problems, even when the flow is dominated by the fluid inertial forces. For instance, resolving the viscous shear stress maps on the surface of fin models might be informative in problems related to flow sensing in the skin of fish [111]. In other words, despite the lower order of magnitude of the measured viscous tangential stress, these components may be of special interest for certain biological applications, which is why we retained them in our analysis.

Vast effort has been dedicated in refining PIV/PTV techniques to research complex flow phenomena in boundary layers, including recirculating zones in cavities [69], cavitation [71], pressure fluctuations [78, 98] and attached/separated layers over obstacles [150]. In the present work, the focus is on providing an estimate of both hydrodynamic stress contributions (shear stress and pressure) from instantaneous captures of the three-dimensional flow field, which requires a compromise between the size of the measurement volume and the vector field spatial resolution. In a recent study, we showed that it is possible to resolve the velocity field inside the shear layer forming over a flat plate pitching in a similar flow regime using the same volumetric PTV experimental setup [111]. In that case, the boundary layer thickness, defined as the distance at which the velocity reaches 99% of the free-stream value, varied dynamically between ∼2 and 6 mm. In the case of our flexible hydrofoil, a rough estimate of the boundary layer thickness can be calculated from the solution for a laminar layer over a flat plate [151]:

$$\begin{array}{c} \delta =5\sqrt{\frac{\nu x}{U}} \end{array}$$ (11)

The value of *x* is the distance along the proximo-distal axis measured from the foil leading edge. The stress maps and curves presented in section 3.1 correspond to locations *x* on the fin in the range of [0.25*L*, *L*], where *L* is the total length of the foil. Even for the thinnest boundary layer, which should occur in exp. 2 where the average velocity magnitude is the largest, the boundary layer thickness evaluated by Eq 11 lies in the range of [2.3,4.6] mm, which is at least three times larger than the vector field spatial resolution (0.75 mm). Despite potential transitions to turbulence due to the oscillation of the flexible membrane, it is reasonable to assume that this thickness will remain in a similar range as that measured on the pitching flat plate [111], thus allowing the estimation of velocity gradients inside the shear layer. Additionally, the virtual surface distance (0.725 mm) is small enough compared to the boundary layer thickness so that the measured tangential stresses can reflect the trends inside the shear layer [110].

This empirical approach allows the estimation of all hydrodynamic force contributions from a single time series of three-dimensional flow fields, thus offering a relevant approach for problems requiring simultaneous shear stress and pressure determination, for example, on the surface of a fish body or fin whose boundary layer thickness is of similar order of magnitude as in the present case [108]. State of the art experimental approaches for volumetric measurements in 3D unsteady flows are constantly evolving with new developments in both hardware and processing algorithms [116, 117, 152–155]. Future technical developments providing increased spatial resolution should allow a more precise estimate of the velocity spatial gradients inherent to the viscous shear stress calculation, while preserving a measurement volume large enough to prescribe proper boundary conditions for the pressure gradient integration approach.

### 2.5 Euler-Bernoulli beam theory

As a comparison, Euler-Bernoulli beam theory is applied in exp. 1 to derive the transversal force distribution on the synthetic fin. This framework requires specific assumptions: that the deflections remain small (*θ*_0_ = 10° at the peduncle), the cross-sections do not deform and remain perpendicular to the deforming axis, and the deformations are confined in the proximo-distal direction. Under these assumptions, the hydrofoil obeys a linearized Euler-Bernoulli equation of motion, where the force per unit length normal to the surface (*f*_*n*_) is given by [43]:

$$\begin{array}{c} \begin{array}{c} f_{n}\left(x,t\right)=m\left(x\right)\frac{\partial^{2}h}{\partial t^{2}}+\frac{\partial^{2}}{\partial x^{2}}(EI\left(x\right)\frac{\partial^{2}h}{\partial x^{2}})+\alpha \frac{\partial h}{\partial t}+\eta \frac{\partial }{\partial t}(\frac{\partial^{4}h}{\partial x^{4}}) \end{array} \end{array}$$ (12)

Despite the fact that a quadratic function offers a good fit to the deflection profiles as described above, for a comparison with Euler-Bernoulli theory with stress-profiles another function *h*(*x*, *t*) has to be used. This is because a quadratic function would lack a non-zero fourth order spatial derivative and hence would not show a force profile. Therefore the time-dependent deflections of the hydrofoil were once more fitted based on the raw images, but this time, with a function inspired by the general solution for a freely vibrating beam, clamped at one end, which is given by:

$$\begin{array}{c} \begin{array}{c} h\left(x,t\right)=C_{1}sin\left(\frac{2\pi t}{T}+\phi_{1}\right)sinh\left(\beta x\right)+C_{2}sin\left(\frac{2\pi t}{T}+\phi_{2}\right)sin\left(\beta x\right) \end{array} \end{array}$$ (13)

The first term on the right hand side of Eq 12 is the inertial force, the second term, the elastic restoring force, the third term, the linear fluid damping and the fourth term, the internal viscoelastic damping, with *m*(*x*), the foil density per unit length, *E*, Young’s modulus, *I*(*x*), the second moment of area, *α* and *η*, the fluid and internal viscous damping coefficients per unit length, respectively. The foil thickness *d*, its width *B*(*x*) and its density *ρ*_*f*_ are used to evaluate *m*(*x*) and *I*(*x*):

$$\begin{array}{c} I\left(x\right)=\frac{B\left(x\right)d^{3}}{12}\;m\left(x\right)=\rho_{f}B\left(x\right)d \end{array}$$ (14)

The boundary condition at *x* = 0, where the pitching motion *θ*(*t*) is imposed, can be expressed as:

$$\begin{array}{c} h\left(x=0,t\right)=0\;\frac{\partial h}{\partial t}\left(x=0,t\right)=\theta \left(t\right) \end{array}$$ (15)

The boundary conditions at the free tip are not explicitly taken into account, since rather than solving Eq 12 for *h*(*x*, *t*), we use the known deflection to calculate the time-dependent load on the fin.

The fluid damping coefficient is determined based on the decay exponent *γ* obtained from the impulse response tests (Eq 1):

$$\begin{array}{c} \alpha =\gamma \rho_{f}B\left(x\right)d \end{array}$$ (16)

This yields values of 0.009 kg m^-1^s^-1^ in air and 0.03 kg m^-1^s^-1^ in water, with a ratio of roughly 3.3 between both. An approximate ratio of 20 was found between *α*_*water*_ and *α*_*air*_ in a previous study, using a similar setup and a polysiloxane foil (with comparable thickness but larger area and larger Young’s modulus) [43]. Based on a modal analysis of the beam deflection, a mathematical relation can be established between *η*, *α*, *γ* and *f*_0_ (see equations B3 and B4 in [43]). Inserting our measured values of *f*_0_ and *γ* (in air) into that equation, we estimated an internal damping coefficient (*η*) for our PDMS fin of the order of 10^-10^ Nm^2^s. The internal damping contribution in Eq 12 is thus expected to be negligible compared to the other terms.

### 2.6 Control volume analysis

A control volume analysis is applied to measure the forces acting on the fluid affected by the fin motion [26, 27, 35, 50, 56, 59, 156] and validate the consistency of the stress distributions obtained on the fin surface. The total force exerted by the fin on the fluid can be expressed as the rate of change of fluid momentum inside the control volume, summed with the hydrodynamic stress tensor projected on the surface of that domain [54, 55, 157]:

$$\begin{array}{c} \vec{F}=\underset{V}{\;\int \int \int \;}\frac{\partial (\rho \vec{u})}{\partial t}dV+{∯}_{S}\left(\hat{n}\cdot \vec{u}\right)\rho \vec{u}dA-{∯}_{S}\hat{n}\cdot sdA \end{array}$$ (17)

We used this approach as a complementary calculation to derive the time-dependent total force and verify the coherence of the PTV-based stress data. The control volume was defined as the whole 3D measurement domain, except the last two grid planes on each outer boundary walls.

### 2.7 Uncertainty analysis

A central point of our work is the evaluation of uncertainties on the hydrodynamic stresses, to ensure that stress maps can be measured accurately on the surface of a small synthetic fin with biologically relevant kinematics. To determine the positional uncertainty arising from the triplet reconstruction scheme, we consider the coordinates of a particle match, where the physical coordinates *X*_*i*_ of the dewarped particles images are averaged over the different cameras *N*_*cam*_. From the uncertainty of each *X*_*i*_ given by several parameters but dominated by the least square errors (*LSE*) from the calibration and the 2D Gaussian fit during the particle identification step, we can then obtain an uncertainty in position *σ*_*x*_ using error propagation given by: $\sigma_{x}=\sigma_{y}=\frac{LSE}{\sqrt{N_{cam}}}≃3.6\mu m$

The uncertainty in the *z*-direction (*σ*_*z*_) is computed using the reference plane distance from the face plate of the cameras mount (∼475 mm) and the distance between the apertures for a half-angle of about 6.5°, leading to *σ*_*z*_ ≃ 32*μ*m. The positional uncertainty propagates to the velocity components:

$$\begin{array}{c} \sigma_{u_{i}}=\frac{\sqrt{2}\sigma_{i}}{\delta t} \end{array}$$ (18)

The time delay *δt* between two laser pulses is considered as exact, resulting in $\sigma_{u_{x}}=\sigma_{u_{y}}≃0.002\;\text{m}/\text{s}$ and $\sigma_{u_{z}}≃0.018\;\text{m}/\text{s}$. During the velocity interpolation onto a regular 3D grid, appropriate filters were applied (see section 2.1). Therefore, these uncertainties are a conservative overestimation.

Noise propagation from the velocity field to the material acceleration and to the integrated pressure field has been the object of many studies [69, 96, 100, 103]. Based on statistical arguments, a normal probability distribution can be determined for the pressure uncertainty, assuming a Lagrangian material acceleration, a normal probability distribution for the velocity uncertainty $N(\mu_{u}=0,\sigma_{u}^{2})$ and integration paths covering *n* nodes [82]:

$$\begin{array}{c} N(n\rho \mu_{u}\Delta x\Delta t^{-1},n\rho^{2}\sigma_{u}^{2}\Delta x^{2}\Delta t^{-2}/2) \end{array}$$ (19)

Accordingly, the pressure error in the case of a zero-expectation value (*μ*_*u*_ = 0) should not exceed the standard deviation:

$$\begin{array}{c} \sigma_{p}⩽\sqrt{\frac{n}{2}}\frac{\rho \sigma_{u}\Delta x}{\Delta t} \end{array}$$ (20)

The Eulerian method would produce less clear error distributions due to the nonlinear advective term of the material acceleration in Eq 7, making it hard to define the uncertainty without *a priori* knowledge of the flow velocity field [82]. The Lagrangian approach is preferable to the Eulerian one as far as it allows a more direct treatment of the measurement parameters in the context of noise reduction. It was also demonstrated in other studies that the Eulerian approach suffers more from the velocity error propagation than the Lagrangian one, especially for flow fields characterized by strong advection velocity [97].

The main sources of uncertainty on the Lagrangian material acceleration are the truncation error associated to the numerical discretization, the precision error propagated from the velocity data and the pseudo-tracking scheme inaccuracy [97, 103, 158]. The discrepancy between the true and estimated particle positions ${\vec{x}}_{p}$ and ${\vec{x}}_{p}^{*}$ in the Lagrangian pseudo-tracking approach results in a discrepancy between the real and approximated velocities ${\vec{u}}_{p}$ and ${\vec{u}}_{p}^{*}$ which adds up to the measurement uncertainty:

$$\begin{array}{c} \sigma_{u_{i}^{*}}^{2}=\sigma_{u_{i}}^{2}+{\left(\left({\vec{u}}_{p}^{*}-{\vec{u}}_{p}\right)\cdot {\hat{x}}_{i}\right)}^{2} \end{array}$$ (21)

$$\begin{array}{c} {\vec{u}}_{p}^{*}-{\vec{u}}_{p}≃(\left({\vec{x}}_{p}^{*}-{\vec{x}}_{p}\right)\cdot \vec{\nabla })\vec{u} \end{array}$$ (22)

Eqs 21 and 22 can be simplified by using the following approximation:

$$\begin{array}{c} \left({\vec{x}}_{p}^{*}-{\vec{x}}_{p}\right)\cdot {\hat{x}}_{i}≃\sigma_{u_{i}}\Delta t \end{array}$$ (23)

$$\begin{array}{c} \begin{array}{c} \Rightarrow \left({\vec{u}}_{p}^{*}-{\vec{u}}_{p}\right)\cdot {\hat{x}}_{i}=\Delta t\left({\vec{\sigma }}_{u}\cdot \vec{\nabla }\right)u_{i}\\ \text{with}\;{\vec{\sigma }}_{u}=\left(\sigma_{u_{x}},\sigma_{u_{y}},\sigma_{u_{z}}\right) \end{array} \end{array}$$ (24)

A forward difference scheme is used to evaluate the material acceleration based on ${\vec{u}}_{p}\left(t\right)$ and ${\vec{u}}_{p}^{*}\left(t+\Delta t\right)$, yielding a total uncertainty on the material acceleration (with *a*_*i*_ = *Du*_*i*_/*Dt*):

$$\begin{array}{c} \sigma_{a_{i}}^{2}=\frac{\sigma_{u_{i}}^{2}+\sigma_{u_{i}^{*}}^{2}}{\Delta t^{2}}=\frac{2\sigma_{u_{i}}^{2}}{\Delta t^{2}}+{\left(\left({\vec{\sigma }}_{u}\cdot \vec{\nabla }\right)u_{i}\right)}^{2} \end{array}$$ (25)

It contains the error propagated from the velocity field and a second contribution from the path line reconstruction inaccuracy, whereas the truncation error from the interpolation of the velocity data, associated to the spatial resolution, was considered negligible compared to the other terms.

The uncertainty on the pressure is proportional to the uncertainty on the material acceleration, the spatial resolution Δ*x*_*i*_ and the number of nodes *n* crossed along the integration path. Moreover, the algorithm involves a median polling among a collection of eight paths. Since it is impossible to predict which direction will correspond to the selected median, averaging the uncertainty in all three directions is a reasonable approach. Besides, given a variable *X* with a statistical sample of size *N* and a median value $\tilde{X}$, a mathematical relation between the variances is obtained [159]:

$$\begin{array}{c} \sigma_{\tilde{X}}=\sqrt{\frac{\pi }{2(N-1)}}\sigma_{X} \end{array}$$ (26)

This yields an approximation for the pressure uncertainty (with the indices 1,2,3 referring to the three cartesian directions):

$$\begin{array}{c} \Rightarrow \sigma_{p}=\frac{\rho }{3}\sqrt{\frac{\pi }{14}}\sum_{i=1}^{3}n\Delta x_{i}\sqrt{\frac{2\sigma_{u_{i}}^{2}}{\Delta t^{2}}+{\left(\left({\vec{\sigma }}_{u}\cdot \vec{\nabla }\right)u_{i}\right)}^{2}} \end{array}$$ (27)

The path length *n* ⋅ Δ*x*_*i*_ typically covers half of the domain size, which is of the order of 20 mm in the *x*-*y* plane, and 8 mm in the *y*-*z* plane. The spatial dependence of pressure errors is analyzed in section 3.4.

The discrete formulation of the viscous shear stress (Eq 5) is associated to an uncertainty:

$$\begin{array}{c} \sigma_{\tau_{ij}}≃\frac{\mu }{\Delta x}\sqrt{\frac{\left(\sigma_{u_{i}}^{2}+\sigma_{u_{j}}^{2}\right)}{2}} \end{array}$$ (28)

Furthermore, the uncertainties are reduced by an additional factor of $\sqrt{N_{time}\times N_{spatial}}$, where *N*_*time*_ is the number of time frames involved in the period-averaging step and *N*_*spatial*_ is the number of grid points used in the spatial average. For the instantaneous stress curves of exp. 1, spatial averaging is performed over the most distal portion (15%) of the fin sides, covering the last four rows of points (*N*_*spatial*_ = 4), whereas the stress curves extracted on the tip surface are obtained by averaging over the three rows of points covering that surface (*N*_*spatial*_ = 3). In the case of the period-averaged distributions, *N*_*spatial*_ = 4, as each stress curve is averaged over the left and right sides and over the dorsal and ventral portions of the foil.

Finally, the pressure and velocity uncertainties are used to estimate the error on the total force obtained from the control volume analysis, based on a discrete version of Eq 17, yielding the error bars of Fig 13.

### 2.8 Temporal and spatial resolutions

The time step between subsequent particles images (*δt*) should be fixed so that the particles do not move over more than ∼10 pixels during that time in the 2D captures (recommendation for the V3V setup from TSI Inc.). This ensures that the particles remain traceable by the algorithm, while limiting the error propagation to the velocity field. In our case, *δt* was set to 2.5 ms to respect that constraint. In comparison, the characteristic time scale of the flow can be estimated based on the characteristic length of our experiment (*a*, the transversal displacement of the fin during a full cycle, 11-14 mm) and the characteristic flow velocity (*U*_*ref*_, the approximate velocity magnitude of a vortex shed by the fin, ∼0.075 m/s), yielding a value of about 0.17 s. Indeed, in our experiments, the characteristic flow structures are vortices generated by the flapping foil and an estimate of *U*_*ref*_ can be obtained by the examination of 2D slices from instantaneous velocity fields, of which an example is illustrated in the left panel of Fig 6.

A similar twofold effect is associated with Δ*t* and Δ*x*, which must remain small enough to permit the Taylor expansions inherent to the calculation of the viscous shear stress and Lagrangian acceleration, but not too small to cause unreasonably large errors propagated from the velocity field. Additionally, the reliability of the pseudo-tracking approach underlying the Lagrangian acceleration can become an issue in the case of strongly curved trajectories involved in highly rotational fluid [69, 103]. The simple criterion Δ*t* ⩾ Δ*x*/*U*_*ref*_ was suggested [82]. The velocity magnitude of the characteristic flow structures is of the order of 0.075 m/s, implying a ratio Δ*x*/*U*_*ref*_ of about 0.01 s for exp. 1 (it is even smaller for exp. 2 where the vortical motion is stronger). Therefore, the condition stated above is met with a temporal resolution of Δ*t* = 0.0125 s. Another criterion for vortex dominated flows was proposed, stating that a vortex should not execute more than half a turn during Δ*t*, with a recommended a ratio of acquisition frequency (*f*_*acq*_ = 1/Δ*t*) to turnover frequency of at least 10 [97]. In our study, the acquisition frequency is 80 s^-1^ and the turnover frequency can be defined as ${\vec{u}}_{ave}/a$, yielding values of 1.4 s^-1^ and 2.07 s^-1^ for exp. 1 and 2, respectively, which shows that the criterion is fulfilled.

For the *queen2* algorithm, pressure errors in the range of 5-10% were reported for Δ*x* < 0.0625*a*, where *a* is the characteristic dimension [77]. The flapping foil in our experiments covers a displacement of 11-14 mm, which translates into a ratio of Δ*x*/*a* between 0.054 and 0.068, indicating that the grid spacing is small enough to produce reasonably small pressure errors. Lastly, a recommendation was made to ensure the validity of the null-pressure boundary condition inherent to the *queen2* algorithm, based on the condition *H*/*a* ⩾ 2 where *H* is half of the domain size [77]. Substituting the tip excursion amplitudes (11-14 mm) for the characteristic dimension *a* and replacing *H* by the average half domain size in the *x* and *y* directions, we conclude that the size of the measurement domain lies right above that limit.

## 3 Results

### 3.1 Hydrodynamic stress distributions

#### 3.1.1. Experiment 1

The parametrized fin deflections are presented in Fig 4 (right panel), at eight selected time points for which the stress distributions are presented. The most proximal portion of the fin is excluded from the 3D particle position field (Fig 4, left panel), and in the following graphs, the zero of the *x* axis is relocated at the initial point on the fin where the flow quantities are extracted.

**Fig 5:**
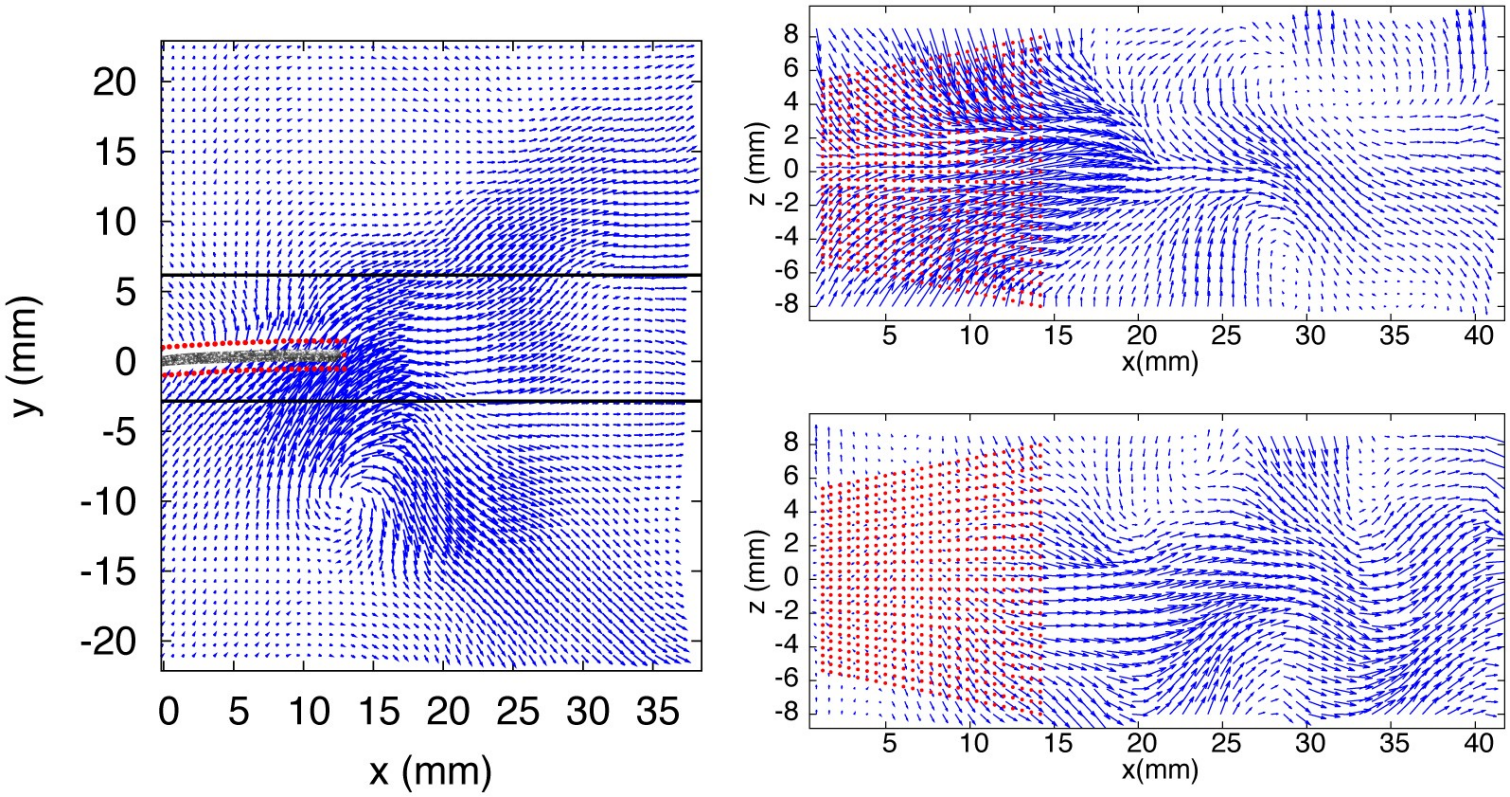
Velocity vectors field—Exp. 1, *t*_0_. *Left panel*: *x* − *y* midplane, with image of the real fin superimposed inside the vitual boundary (red points). *Right panel*: *x* − *z* planes indicated by black lines in the left panel.

**Fig 6:**
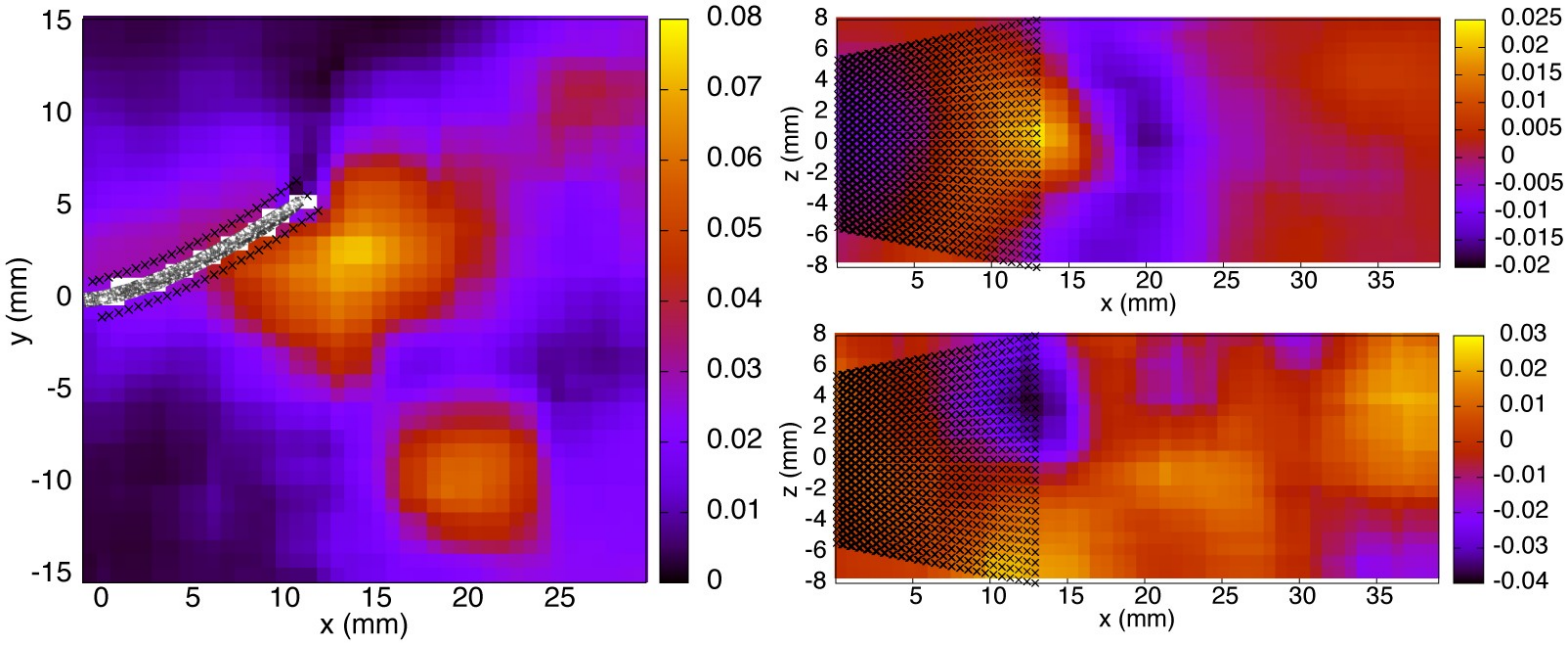
Velocity color maps (m/s)—Exp. 1, *t*_1/4_. *Left panel*: Velocity magnitude in the *x* − *y* midplane. *Right*, *top and bottom panels*: *y* and *z* velocity components, respectively, in a *x* − *z* plane below the foil (*y* = -5.275 mm).

The velocity field at *t*_0_ is shown in Fig 5, where 2D slices from the measurement volume are presented: one *x* − *y* plane (in the middle of the domain) and two *x* − *z* planes located on both sides of the fin. In all planes, the shedding of counterrotating vortices can be observed, corresponding to the 2D projections of vortex rings. In Fig 6, the velocity field at *t*_1/4_ is represented by a color map. The velocity magnitude is depicted in the *x* − *y* midplane, where high values are associated to two vortices, one previously shed in the downstream wake and a second still in formation, attached to the foil. The vertical (*u*_*y*_) and transversal (*u*_*z*_) velocity components in a single *x* − *z* plane are also shown in Fig 6.

**Fig 7:**
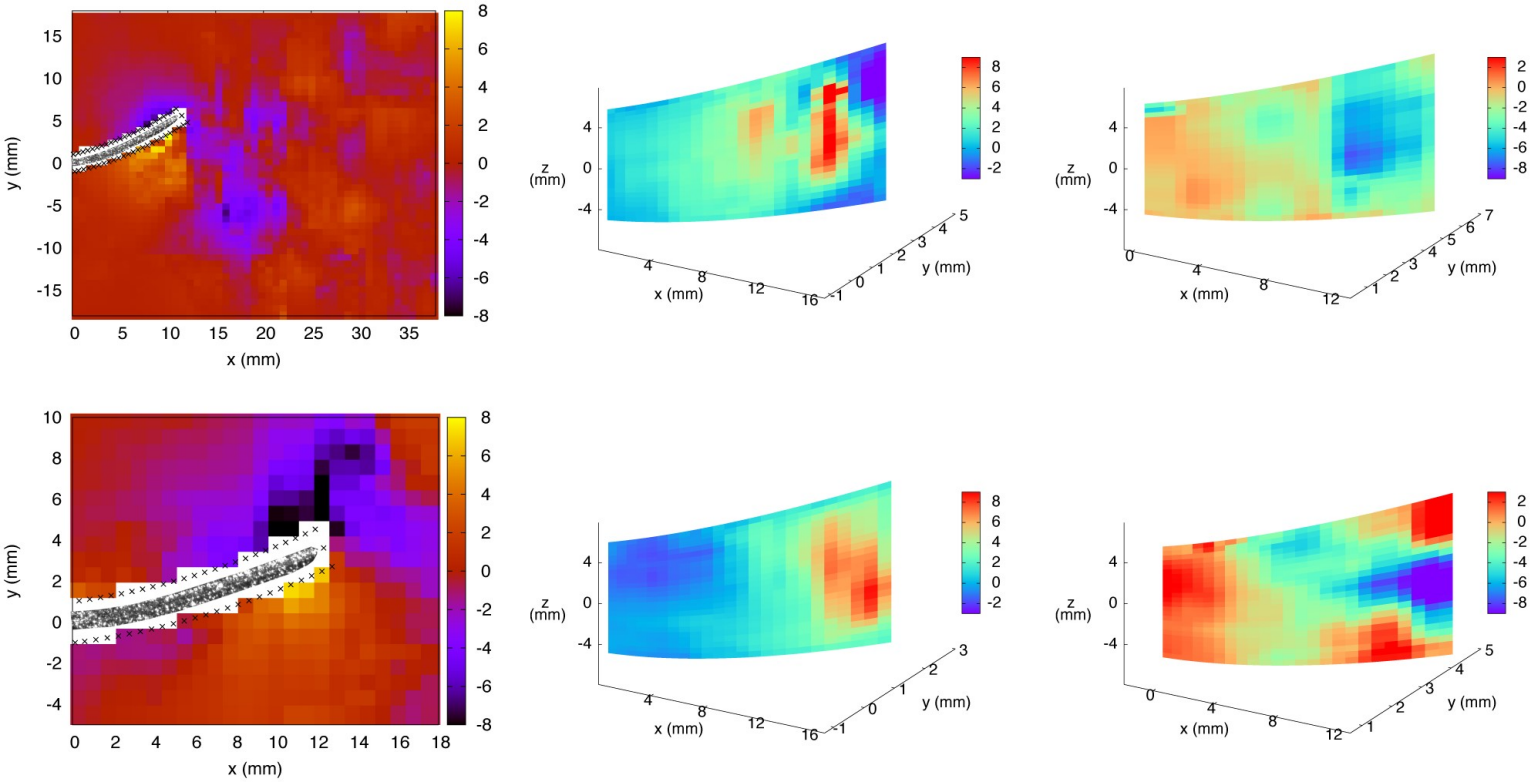
Pressure color maps (Pa) from exp. 1, in the *x* − *y* midplane (left column) and on the left and right sides of the fin (middle and right columns respectively), at *t*_1/4_ (top row) and *t*_3/8_ (bottom row).

A pair of subsequent time points (*t*_1/4_ and *t*_3/8_) is analyzed to ensure that the stress profiles on the fin surface present a realistic time-evolution (no unphysical abrupt transitions). Fig 7 (left column) offers an overview of the pressure distributions in the *x* − *y* midplane extracted from the 3D domain. The fin is traveling towards negative *y* values. A vortex is visible close to the location (*x* = 17.5mm, *y* = −7.5mm), associated with a core of negative pressure. We are especially interested in the stress maps directly on the fin surfaces. Fig 7 (middle and right columns) presents the pressure distributions on the left and right sides of the foil (at *t*_1/4_ and *t*_3/8_). The left side of the foil at *t*_1/4_ is subjected to positive pressure, yielding a normal stress directed towards its surface. The maximum value is of the order of 10 Pa, located distally on the fin, although not at the very end, and centrally along the dorso-ventral axis. The tip corners experience negative pressure with values around -4 Pa. A similar trend is observed for the left side in the following time frame (*t*_3/8_), where the pressure maximum is reduced to a value around 8 Pa. At *t*_1/4_, the right side of the fin is subjected to negative pressure on its most distal portion, of the order of -7 Pa, an effect that concentrates more centrally along the dorso-ventral axis at *t*_3/8_, with a minimum decreasing to approximately -15 Pa, along with the appearance of two small positive pressure spots of about 5 Pa close to the dorsal and ventral corners.

**Fig 8:**
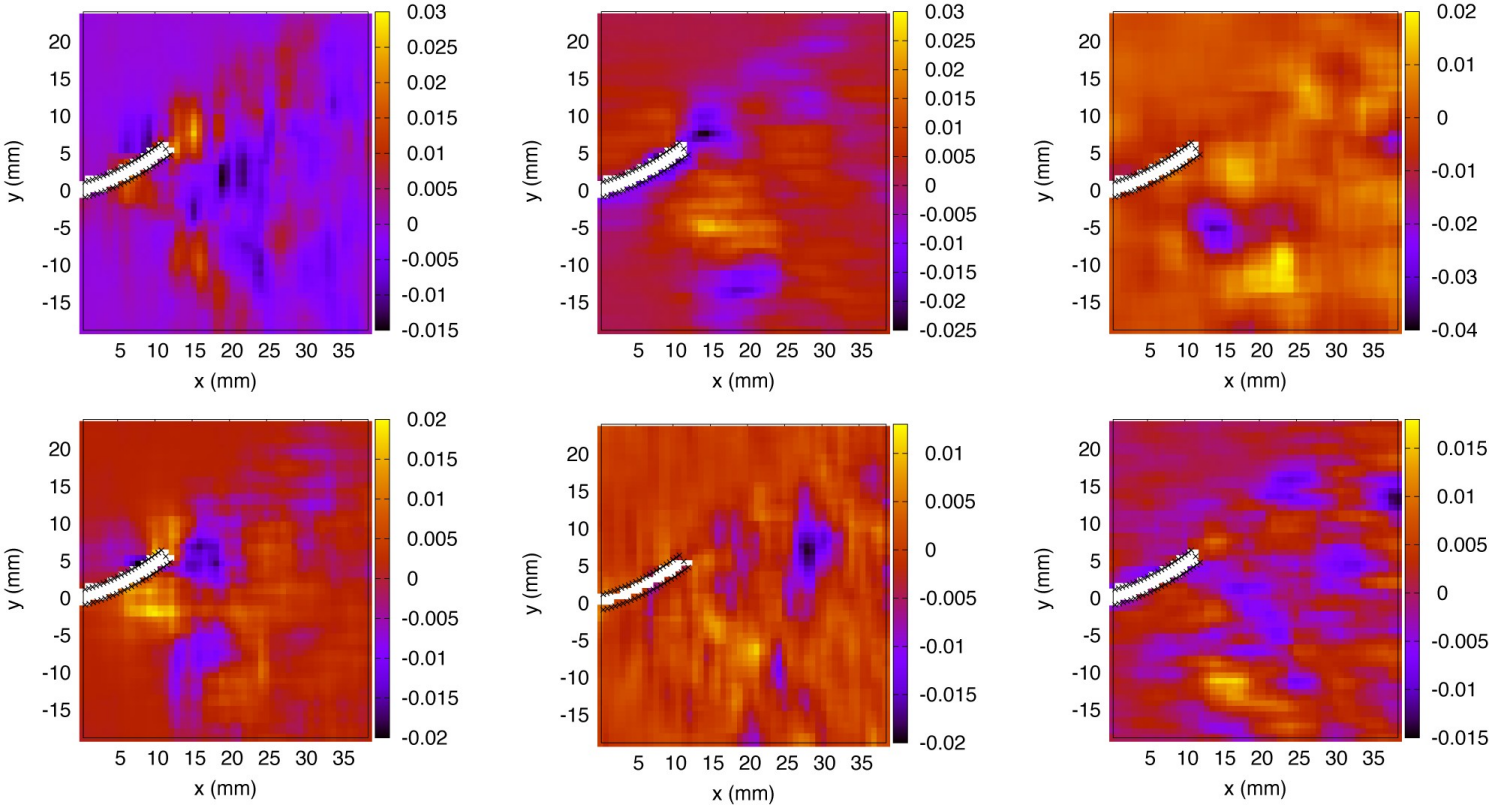
Six components of the viscous stress tensor from exp. 1, at *t*_1/4_, in the *x* − *y* midplane (colorbar in Pa). From upper left panel to bottom right panel: *τ_xx_*, *τ_yy_*, *τ_zz_*, *τ_xy_*, *τ_xz_* and *τ_yz_* (see Eq 4).

The relative contribution of the viscous stress within the total hydrodynamic stress, in comparison with the pressure, can be estimated based on the distributions of the six components *τ*_*ij*_ from Eq 4, namely, the viscous stress acting in the direction *i* on a surface perpendicular to *j*. These six components are presented in the *x*-*y* midplane for *t*_1/4_ in Fig 8. As expected for Reynolds numbers corresponding to the inertial flow regime, these distributions are about two orders of magnitude lower as compared to the pressure. Local variations on the virtual fin surfaces are visible, corresponding to the viscous shear profiles within the boundary layer. As explained in section 2.4, the cartesian components (*τ*_*xx*_, …, *τ*_*xz*_) are subsequently projected on the surface unit vectors and decomposed into proper components in the reference frame of the fin (proximo-distal, dorso-ventral, left-right). Apart from these noticeable variations in the vicinity of the solid surface (especially for *τ*_*xx*_, *τ*_*yy*_ and *τ*_*xy*_), special flow signatures are observed within the vortex that was identified from its negative pressure core in Fig 7, including paired regions of opposite-signed viscous stress, typical of the rotational nature of the vortex. For an incompressible fluid such as water, the divergence-free condition ($\vec{\nabla }\cdot \vec{u}=0$) implies that the sum of *τ*_*xx*_, *τ*_*yy*_ and *τ*_*zz*_ should be equal to zero. This condition was verified for the instantaneous flow field presented in Fig 8, where the divergence of the velocity vector field was found to be within ±15 s^-1^, with an average over the whole volume (in absolute values) of 2.4 s^-1^. The uncertainty on the divergence of $\vec{u}$ can be evaluated using error propagation from the velocity components, yielding a value of 17.2 s^-1^ (with centered finite differences). Therefore, the measured flow field is divergence-free within experimental uncertainties.

**Fig 9:**
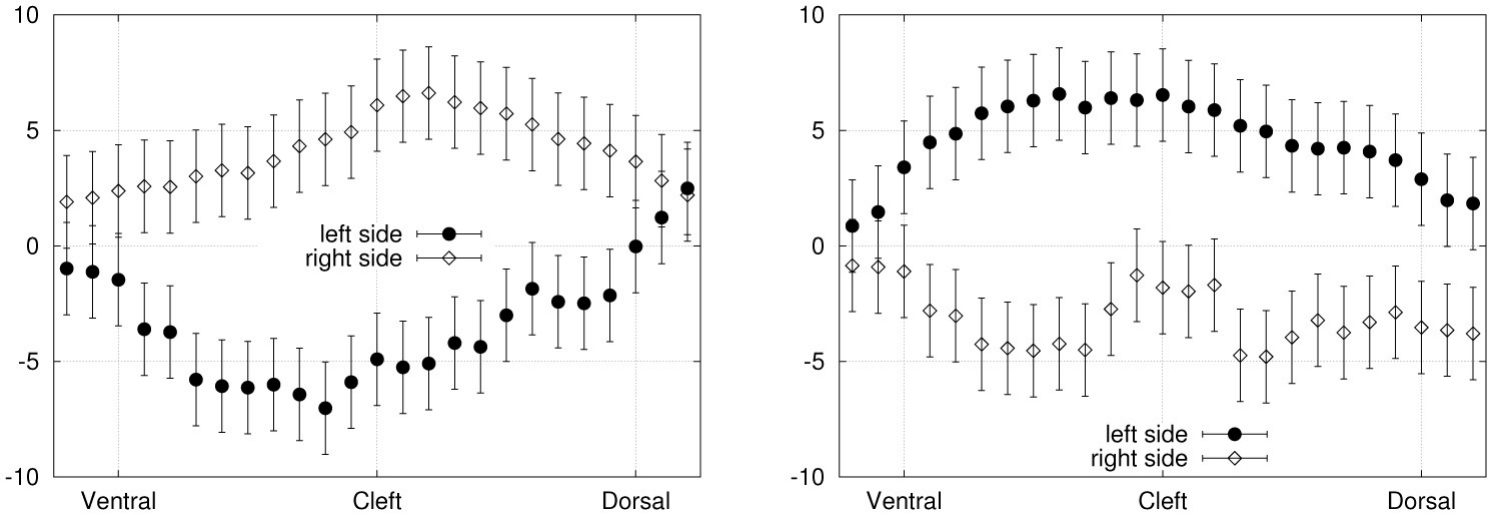
Exp. 1—Normal stress component (Pa), averaged over the most distal portion (15%) of the left and right sides surfaces, plotted against the dorso-ventral axis. *Left panel*: *t*_1/4_. *Right panel*: *t*_3/4_.

The examination of instantaneous stress maps at mirroring time points (equivalent motion instants such as *t*_0_ and *t*_1/2_ or *t*_1/4_ and *t*_3/4_) is used to corroborate the consistency of the symmetrical distributions. Spatial symmetry between the left/right and ventral/dorsal sides of the hydrofoil is also verified to validate the stress maps. The stress patterns are drawn more explicitly by plotting the individual components along the relevant axes. The curves shown in Fig 9 represent the normal stress component (at *t*_1/4_ and *t*_3/4_) plotted against the dorso-ventral axis, averaged over the most distal portion (last 15%) of the membrane. By comparing both panels of Fig 9, we notice that as the fin is passing through mirroring sites, the normal stress on the left and right sides are mirrored. The absolute value of the pressure is maximal at the center of the foil and decreases towards the dorsal and ventral edges. The side which is leading the motion (the left side at *t*_1/4_ and the right side at *t*_3/4_) experiences a negative normal stress, which means that the stress is directed towards the surface. On the the trailing side, the positive normal stress points in the direction of the outward normal, as the fluid tends to hold the foil back. Small discrepancies between the mirroring time points can be attributed to experimental errors perturbing the symmetry of the foil motion, such as an imperfect positioning relative to the incoming flow.

**Fig 10:**
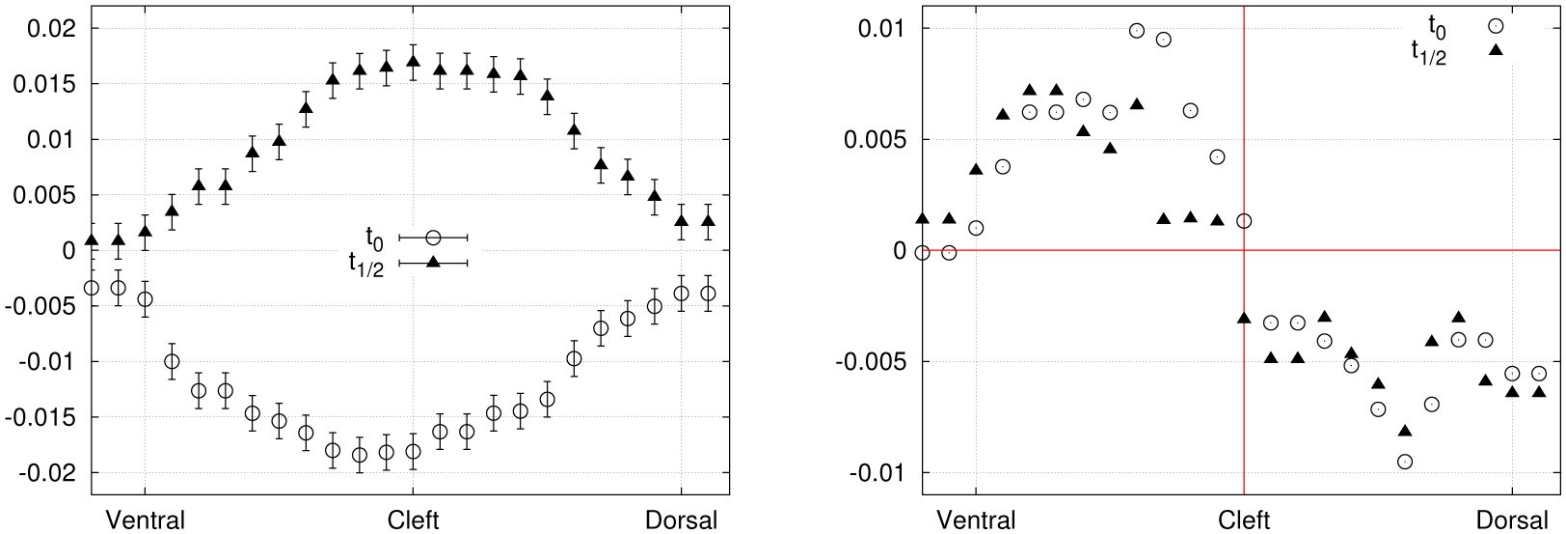
Exp. 1 (*t*_0_ and *t*_1/2_)—Shear stresses (Pa) on the tip surface, plotted against the dorso-ventral axis. *Left panel*: Left-right (transversal) stress component. *Right panel*: Dorso-ventral stress component.

In Fig 10, we consider the viscous shear stresses on the tip surface, at mirroring time points *t*_0_ and *t*_1/2_. At *t*_0_, the fin travels towards its right side (positive *y* values) and the tip surface experiences shear stress pointing in the left direction (*S*_*lr*_ < 0), with a maximum absolute value in the center. The opposite trend is observed at *t*_1/2_. As for the dorso-ventral shear stress, it is positive on the ventral portion of the tip, and negative on the dorsal part, implying that the stress is directed towards the center as the fluid tends to contract the fin tip surface. Naturally, this effect occurs for both *t*_0_ and *t*_1/2_ since the dorso-ventral axis is symmetric between the mirroring time frames.

**Fig 11:**
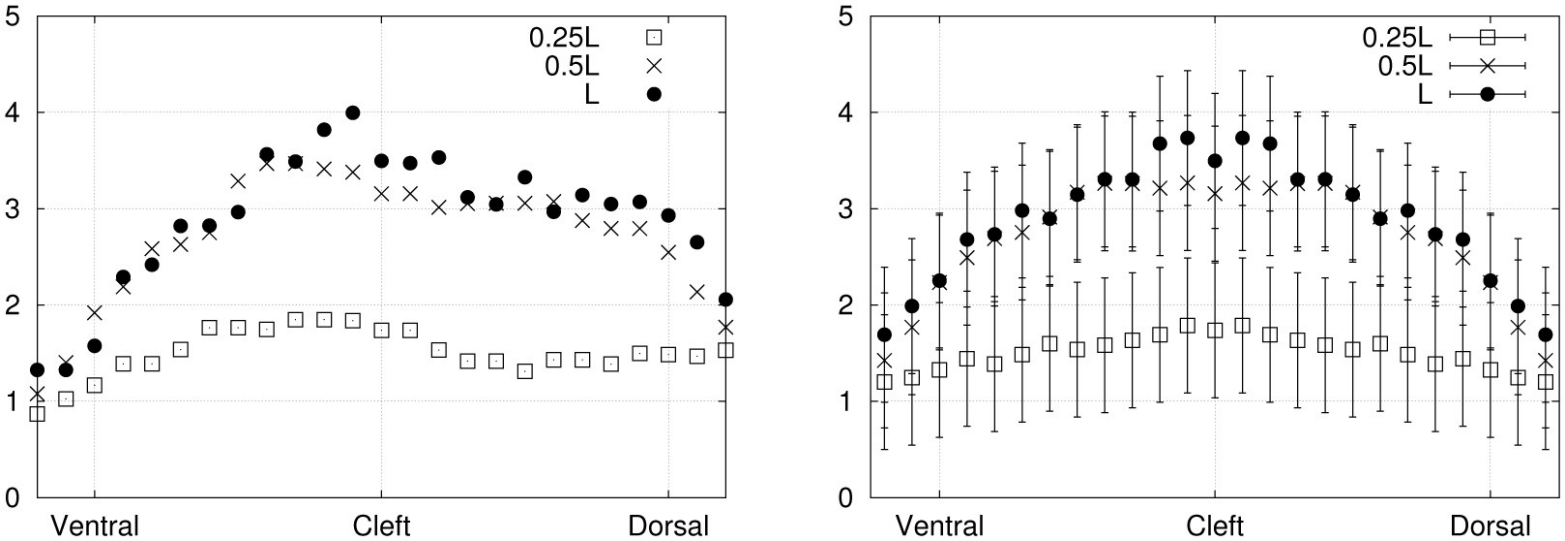
Period-averaged absolute value of the normal stress component (Pa) on the sides surfaces (exp. 1), plotted against the dorso-ventral axis, before and after averaging over both symmetrical sides (dorsal and ventral), in left and right panels respectively.

Finally, the period-averaged absolute value of the normal stress component (averaged over a full cycle) is illustrated in Fig 11. Three curves are shown, each one corresponding to a selected position along the proximo-distal axis of the fin: at 25%, 50% and 100% of the total length (0.25*L*, 0.5*L* and *L*).

#### 3.1.2. Experiment 2

**Fig 12:**
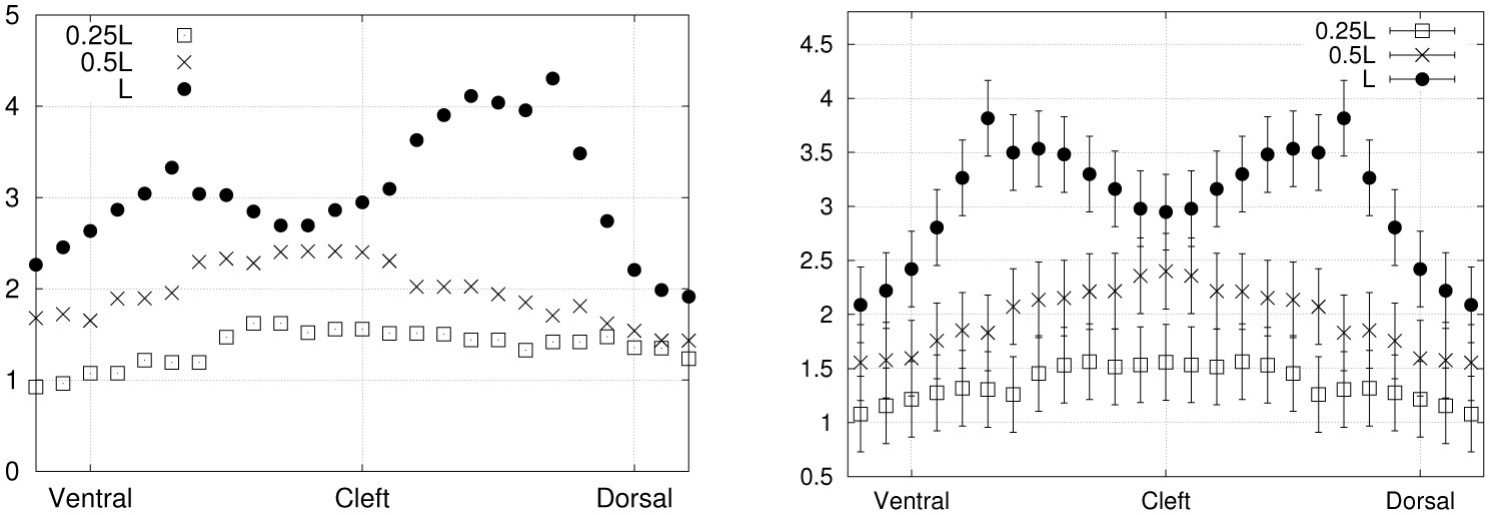
Period-averaged absolute value of the normal stress component (Pa) on the sides surfaces (exp. 2), plotted against the dorso-ventral axis, before and after averaging over both symmetrical sides (dorsal and ventral), in left and right panels respectively.

An interesting application of the hydrodynamic stress mapping method lies in the investigation of particular stress signatures or thresholds along the main axes of the hydrofoil, where the effects of varying specific flow parameters can be analyzed. The period-averaged absolute values of the normal stress are compared between exp. 1 (Fig 11) and exp. 2 (Fig 12) to deduce how the hydrodynamic load is changing over the fin surface depending on the flow conditions (without or without an external current). These distributions correspond to averages over complete cycles. Experimental imperfections in the foil geometry and its positioning inside the flow chamber can result in a slight asymmetry of the fluid force distributions on the dorsal and ventral sides. Averaging the stress curves over both sides allows to reduce noise and experimental errors. For the distributions prior to dorso-ventral averaging, the error bars are not shown for sake of clarity, as they would be larger than for the symmetrical curves by a factor of $\sqrt{2}$.

The magnitude of the normal stress at 50% of the fin length (0.5*L*) is reduced as a consequence of applying an external flow (exp. 2). Besides, at the distal location on the foil (*L*), the curve goes from a single peak for exp. 1 to a curve with two maxima for exp. 2, with similar maximum magnitudes. Therefore, the presence of an external advecting flow, combined with slightly larger flapping amplitude, shifts the stress curves to lower values at specific positions along the fin (about half-way through the proximo-distal axis) and modifies the peaks profile on the most distal surface. These results support the practicality of the stress mapping method as a powerful tool for analyzing the distribution of fluid forces on submerged and deforming objects.

### 3.2 Control volume analysis

**Fig 13:**
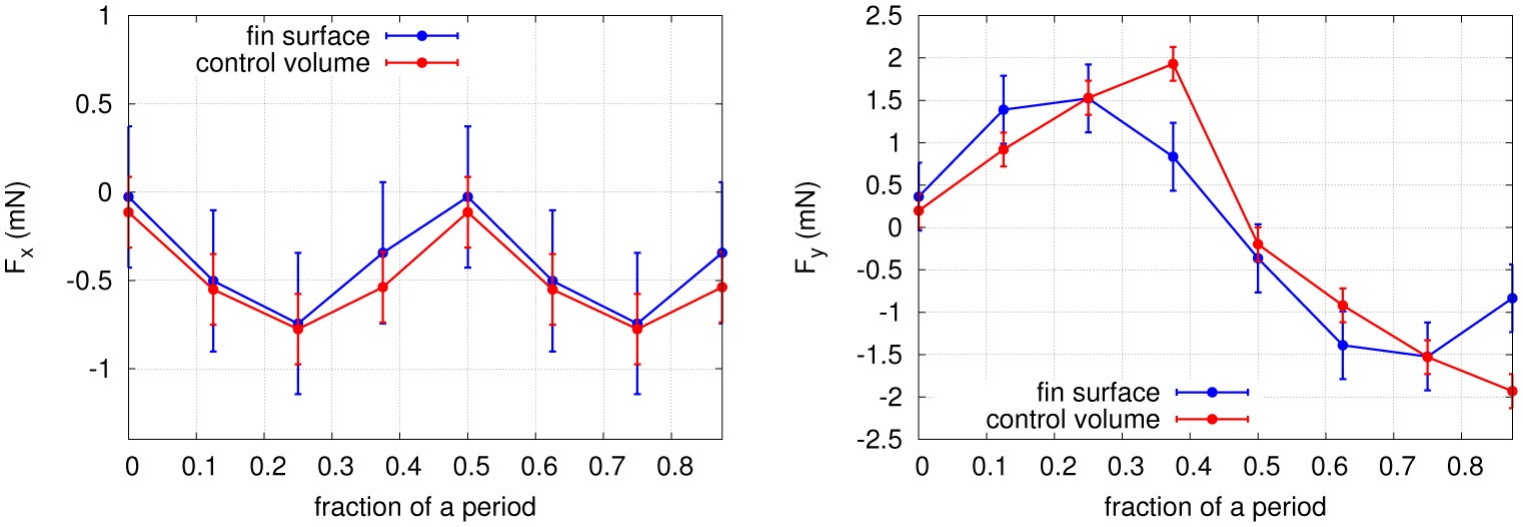
Time evolution of the total force in the *x* and *y* directions, obtained from a control volume analysis (see Eq 17) and from the integration of the PTV-derived stress over the fin surface.

A control volume analysis was used to validate the consistency of the instantaneous force data. The total forces acting on the fin in the *x* and *y* directions (*F*_*x*_ and *F*_*y*_) were calculated from Eq 17, for the eight selected time points of exp. 1. The results are shown in Fig 13. Fair agreement is found between the control volume forces and the values obtained by integrating the stress distributions on the fin surfaces within the uncertainty of the pressure determination corresponding to an uncertainty in the force of *σ*_*F*_ = 0.4 mN for the PTV-integrated results and *σ*_*F*_ = 0.2 mN for the control volume method. Both approaches describe a net force in the negative *x* direction with a maximal magnitude of ∼0.75 mN at mirroring instants *t*_1/4_ and *t*_3/4_ (where the hydrofoil tip excursion is maximal), implying that the foil experiences a net thrust as it is propelled upstream. Both methods also capture a net force in the positive *y* direction in the first half of the period (instants *t*_1/8_ to *t*_3/8_) and in the negative *y* direction in the second half of the period (instants *t*_5/8_ to *t*_7/8_), with a maximal magnitude of ∼1.75 mN. We conclude that the time-evolution of the PTV-based stress distributions is consistent with the fluid forces obtained from a control volume analysis. A small offset is seen in *F*_*y*_ at *t*_3/8_ and *t*_7/8_, which can be attributed to experimental errors in the velocity field. To investigate the source of this offset, we performed again the control volume calculation, using a virtual volume slightly closer to the fin in the *x* and *y* directions (not in the *z* direction though to ensure that the whole fin remains included in the control volume). The results are identical within the uncertainties, indicating that the shift originates from the PTV-based stress integrated over the fin surfaces, and not from the control volume. Globally, the magnitude and shape of the time-resolved total forces are in good agreement between both approaches.

### 3.3 Euler-Bernoulli force distributions

**Fig 14:**
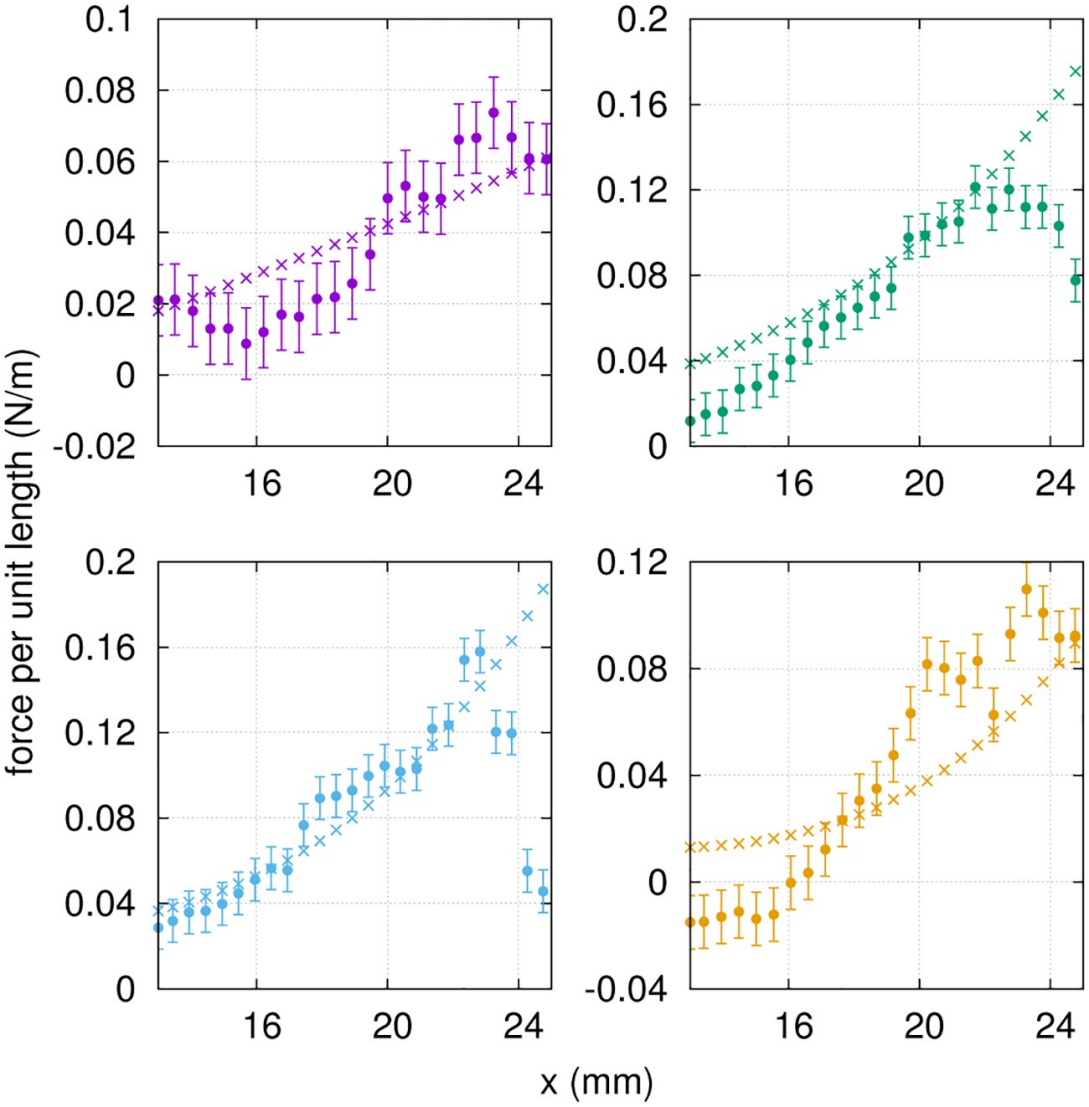
Force per unit length along the proximo-distal axis of the fin (exp. 1), from the PTV-based strees distributions (full circles) and the Euler-Bernoulli load calculation (crosses), at *t*_0_, *t*_1/8_, *t*_1/4_ and *t*_3/8_ from top left to bottom right, respectively. Color code follows that of Fig 4. The error bars for the PTV-based force have been obtaine as described in section 3.4.

The motion instants *t*_0_, *t*_1/8_, *t*_1/4_ and *t*_3/8_ from exp. 1 (covering half a period) were selected to evaluate transversal forces in the Euler-Bernoulli framework. The parametrized deflection of the foil (Eq 13) was used to compute the appropriate derivatives in Eq 12, and to provide an estimate of the transverse force per unit length (pointing upwards) along the proximo-distal axis of the fin. The results are presented in Fig 14, and compared with the force distributions obtained by integrating the PTV-based normal stress distributions over both sides of the hydrofoil. The main contribution to the total transverse load is the elastic restoring force, which is of the order of 0.1 N/m, followed by the inertial force, of the order of 0.01 N/m. The linear fluid damping and the structural damping contributions are negligible in comparison, of the order of 0.001 N/m. The curves from both approaches are consistent in terms of order of magnitude and proximo-distal trends. The force drop at the tip of the fin for *t*_1/8_ and *t*_1/4_, captured by the PTV-based stresses, is not well reproduced by the Euler-Bernoulli model. We attribute this small discrepancy to the larger tip deflections at these specific time points, where the Euler-Bernoulli formalism is less applicable by definition. Moreover, the quality of the fit (Eq 13) is slightly decreased at the tip of the fin for these time points, where the deflection is overestimated by the fitted function. As expected, the Euler-Bernoulli calculation offers a more approximate solution to the force maps, where the hydrodynamic forces obtained directly from the 3D flow field provide more detailed local stress variations. In brief, the force distributions obtained from the Euler-Bernoulli deflection analysis compare reasonably well with the PTV-based normal stresses, given the underlying assumptions.

### 3.4 Uncertainties

From Eq 20, it is possible to provide a raw estimate of the pressure uncertainty, using the parameters specific to our experiments. With an approximate integration path length (covering half the fluid domain) of 30 nodes in the *x* and *y* directions and 10 nodes in the *z* direction, we get an approximate pressure error of 1 Pa (after averaging over the *x*, *y*, *z* directions).

A more detailed analysis is rendered possible by the use of Eq 27. In the case of exp. 1, the error on the material acceleration arising from the Lagrangian path reconstruction inaccuracy (the second term in Eq 25 which carries the spatial dependence) was calculated everywhere in the 3D domain. Spatial average over the whole volume and temporal average over a complete period (using the 8 time frames *t*_0,…,7/8_) yielded the following values:

$$\begin{array}{c} \left\langle \left({\vec{\sigma }}_{u}\cdot \vec{\nabla }\right)u_{x}\right\rangle =0.029m/s^{2}\\ \left\langle \left({\vec{\sigma }}_{u}\cdot \vec{\nabla }\right)u_{y}\right\rangle =0.023m/s^{2}\\ \left\langle \left({\vec{\sigma }}_{u}\cdot \vec{\nabla }\right)u_{z}\right\rangle =0.040m/s^{2} \end{array}$$

The larger value obtained for the *z* component contributes to larger uncertainties accumulating in that direction. In comparison, the first term of Eq 25, which contains the material acceleration uncertainty propagated from the PTV errors, produces values at least one order of magnitude larger:

$$\begin{array}{c} \left\langle \frac{\sqrt{2}\sigma_{u_{x}}}{\Delta t}\right\rangle =0.23m/s^{2}\\ \left\langle \frac{\sqrt{2}\sigma_{u_{y}}}{\Delta t}\right\rangle =0.23m/s^{2}\\ \left\langle \frac{\sqrt{2}\sigma_{u_{z}}}{\Delta t}\right\rangle =2.04m/s^{2} \end{array}$$

**Fig 15:**
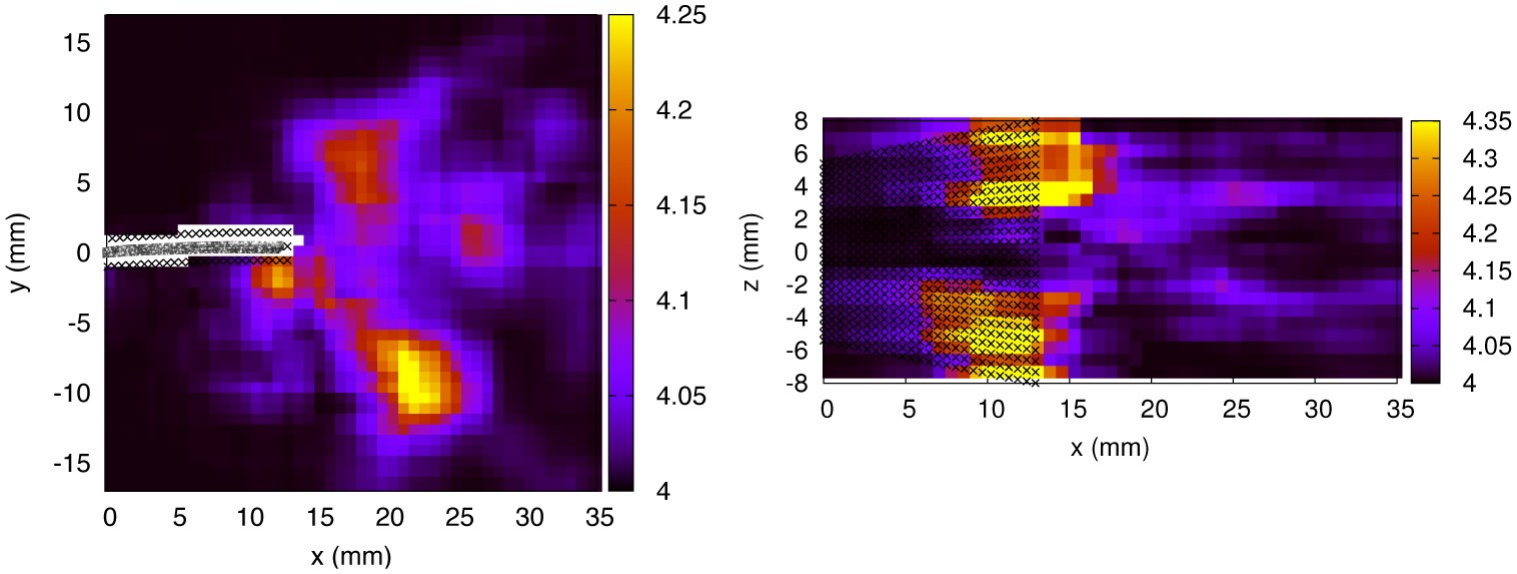
Pressure uncertainty in Pa (exp. 1, *t*_0_). *Left panel*: *x* − *y* midplane. *Right panel*: *x* − *z* plane at *y* = -5.275 mm.

**Fig 16:**
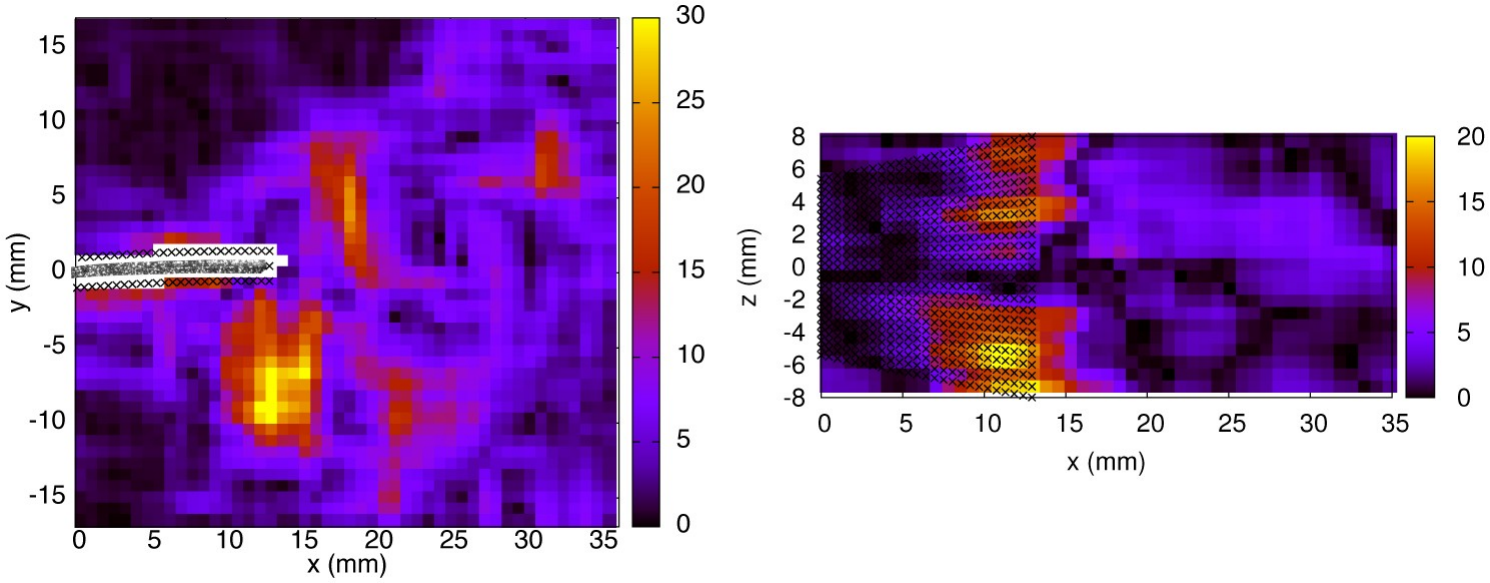
Fluid vorticity in s^-1^ (exp. 1, *t*_0_). *Left panel*: Vorticity magnitude in the *x* − *y* midplane. *Right panel*: Vorticity *x*-component (in absolute value, |*ω_x_*|) in a *x* − *z* plane (*y* = -5.275 mm).

We thus conclude that the error propagated from the velocity field constitutes the main source of error in the pressure calculation, whereas the contribution from the inaccuracy of the Lagrangian path reconstruction is almost negligible. The resulting pressure uncertainty (*σ*_*p*_), averaged over the 3D domain and a complete motion cycle, is equal to 4.03 Pa. The spatial dependence of *σ*_*p*_ is shown in Fig 15 (for *t*_0_, exp. 1). The fluid vorticity ($\vec{\omega }$) and its streamwise component (in absolute value, *ω*_*x*_) are illustrated in Fig 16. The pressure uncertainty is found to be spatially correlated to the vorticity, in particular, the error becomes larger vis-a-vis the tip corners, where high vorticity around the $\hat{x}$ axis is found. This correlation is expected since the spatial dependence of *σ*_*p*_ is carried by the term associated to errors in the pseudo-tracking method. Thus, it is no surprise that the regions where the particles follow paths with strong curvature (high vorticity) are the regions where the Lagrangian path reconstruction, which assumes a linear displacement between subsequent velocity fields, performs more poorly.

Including the reduction factor due to spatial and temporal averaging, the final uncertainties on the normal stress in exp. 1 are *σ*_*p*_≃ 2 Pa for the instantaneous distributions (Fig 9) and 0.7 Pa for the period-average (Fig 11). The uncertainties on the viscous shear stresses (acting on the tip, Fig 10) are *σ_τyx_* ≃ 1.6 mPa for the left-right component and *σ_τzx_* ≃ 10 mPa for the dorso-ventral component. However, the dorso-ventral symmetry and the temporal symmetry in the shear stress curves suggest on the contrary that these stress distributions are well resolved within an uncertainty range that does not exceed 3 mPa. We interpret this as an indication that the velocity errors stated in section 2.7 are slightly overestimated, as they do not include the appropriate error reduction associated to the filtering and smoothing steps. For that reason, the error bars were exceptionally omitted on that specific graph (Fig 10, lower panel). For exp. 2, the error bars in Fig 12 correspond to *σ*_*p*_ ≃ 0.35 Pa (in that case, the period-average was performed over 32 frames).

Finally, the error bars of Fig 14 were calculated based on *σ*_*p*_, the integration path length (the average fin width) and the number of grid points along that path (25 on each side of the fin).

## 4 Discussion and outlook

In the present study of a prototypical fin-like foil, we performed three-dimensional hydrodynamic stress calculations based on a pressure gradient multi-path integration scheme, which we applied to a time series of volumetric PTV measurements of an unsteady vortical flow. This method was shown to produce well resolved stress curves along the biologically relevant axes of the synthetic fin, allowing to gain precise information about the interaction between the deforming surface and its direct fluid environment. We explored in details the error propagation for both the normal and tangential stresses. We validated the results using a control volume analysis and compared the force trends with values determined from the Euler-Bernoulli beam theory.

The non-intrusive approach of hydrodynamic stress mapping constitutes a promising path to deepen our understanding of the relation between the morphometric and elastic properties of hydrofoils and the fluid forces which they experience. We foresee numerous applications to this experimental methodology, in domains such as the engineering of underwater propulsive foils or the investigation of hydrodynamic forces in aquatic animal locomotion. Propulsive foils involved in marine robotic systems would greatly benefit from the employment of small scaled models for which the distribution of hydrodynamic stresses and their time evolution could be described everywhere on the surface, without the need for intrusive mechanical transducers, thus guiding the structural design with precise knowledge of the local mechanical loads. In a parallel train of thought, mapping the stress patterns on the surface of biomimetic synthetic fins can provide new insights about the mechanical constraints under which real fins have evolved to attain their exact morphology.
